# TXNIP upregulation controls metabolism and cell cycle during androgen deprivation therapy in prostate cancer

**DOI:** 10.1038/s41419-025-08128-4

**Published:** 2025-11-10

**Authors:** Sergio Alcon-Rodriguez, Juan C. Mayo, Pedro Gonzalez-Menendez, Iván Fernandez-Vega, David Hevia, Sheila Fernandez-Vega, Alba Moran-Alvarez, Daniela Pineda-Cevallos, Miguel Alvarez-Mugica, Pablo Rodriguez-Gonzalez, Belen Garcia-Soler, Jorge Zamora, Jose M. C. Tubio, Rosa M. Sainz, Isabel Quiros-Gonzalez

**Affiliations:** 1 Department of Morphology and Cell Biology, School of Medicine, University of Oviedo, Oviedo, Spain; 2 Oncology Institute of Principado de Asturias (IUOPA), Oviedo, Spain; 3 Health Research Institute of Principado de Asturias (ISPA), Oviedo, Spain; 4 Department of Pathology, Hospital Universitario Central de Asturias (HUCA), Oviedo, Spain; 5 Biobank of the Principality of Asturias (BioPA), Oviedo, Spain; 6 Department of Physical and Analytical Chemistry, School of Chemistry, University of Oviedo, Oviedo, Spain; 7 Department of Urology, Hospital Valle del Nalón, Langreo, Spain; 8 Mobile Genomes, Centre for Research in Molecular Medicine and Chronic Diseases (CIMUS), Universidad de Santiago de Compostela, Santiago de Compostela, Spain; 9 Instituto de Investigaciones Sanitarias de Santiago de Compostela (IDIS), Santiago de Compostela, Spain; 10 Department of Zoology, Genetics and Physical Anthropology, Universidad de Santiago de Compostela, Santiago de Compostela, Spain

**Keywords:** Cancer metabolism, Metabolomics, Translational research, Prognostic markers, Cancer models

## Abstract

Thioredoxin-Interacting Protein (TXNIP) is an arrestin at the crossroad of redox and glycolytic metabolisms. Prostate cancer (PCa) exhibits a unique metabolic profile due to the glycolytic nature of healthy prostate tissue. We hypothesize that TXNIP plays a pivotal role in the progression of PCa to castration-resistant prostate cancer (CRPC), an incurable stage of the disease characterized by profound metabolic reprogramming and independence from androgens. Only a subset of patients progresses to CRPC, and current stratification tools lack robust biomarkers. TXNIP expression is directly suppressed by androgens and diminishes during tumor initiation and progression, as demonstrated in both human samples and a prostate adenocarcinoma mouse model (TRAMP). TXNIP regulates glucose metabolism by sequestering the glucose transporter GLUT1 away from the membrane, shifting metabolism from glycolysis to glutaminolysis. Nuclear-localized TXNIP induces cell cycle arrest through the upregulation of p27^kip1^ which is downregulated together with TXNIP in CRPC. The response to androgen deprivation therapy (ADT) strongly depends on TXNIP expression. In the murine model, TXNIP levels were significantly higher in ADT responders compared to non-responders. Furthermore, TRAMP-*Txnip*^**−**/**−**^ prostate tumors exhibited a poorer response to ADT, with increased Ki67 and enhanced viability. In clinical samples, all patients on relapse showed low levels of TXNIP and progressed to CRPC. Our findings identify TXNIP as a critical regulator of cell cycle and glucose metabolism in PCa and emphasize for the first time its essential role in mediating therapeutic responses to ADT.

## Introduction

The prostate gland is dependent on androgens and exhibits a unique metabolism due to its role in citrate export for seminal fluid production [1], resulting in TCA cycle block and a glycolytic metabolism [1].

PCa is the fifth leading cause of cancer-related death in men worldwide [2]. ADT has a positive effect on tumor regression alone or combined with chemotherapy or radiotherapy [3]. Despite the effectiveness of ADT, 10–20% of tumors eventually develop resistance, transitioning into CRPC [4], and their survival rate ranges from 5 to 30 months [4].

Citrate export stops during cancer initiation, redirecting glucose into the TCA cycle in the early stages of PCa [1]. Among other survival strategies to meet the increased glucose needs, the insulin-independent glucose transporter GLUT1 (*SLC2A1*) is overexpressed [5, 6], enhancing survival to redox-mediated cell death in androgen-sensitive PCa cells [7]. Additionally, GLUT1 contributes to the metabolic adaptations that support ADT resistance [8]. GLUT1 is overexpressed in many other tumors and is associated with poor prognosis [5].

TXNIP is a protein from the alpha-arrestin family which carries out many known functions in cell biology. Among them, TXNIP is at a crossroad between glucose and redox metabolism by inhibiting glucose uptake by sequestering GLUT1 [9] and thioredoxin (TRX), blocking its antioxidant activity [10] and other redox-independent functions [11]. TXNIP is the most upregulated gene in pancreas by glucose [12]. Other key redox enzymes, such as MnSOD/SOD2, have been associated with androgen-independence [13]. Additionally, TXNIP stabilizes p27^kip1^ to induce cell cycle arrest [14] and cellular senescence [15]. Thus, TXNIP imbalance has been linked to various diseases, including diabetes, ischemia/reperfusion injury, Alzheimer’s disease, and cancer [16], where it has been proposed as a tumor suppressor and biomarker for cancer progression [17].

## Material and methods

### Reagents, cell culture and lentiviral transduction

All reagents, experimental models, companies, and references are listed in Table SI.

Human prostate cancer cell lines LNCaP (androgen-dependent) and PC-3 cell line (androgen-independent) were cultured in RPMI medium and DMEM/F12, respectively. Both were supplemented with 10% FBS, 2 mM L-glutamine, and 1% pen/strep cocktail (+amphotericin for PC-3).

For TXNIP overexpression, control (pLV[Exp]-Puro-EF1A > ORF_Stuffer) and TXNIP cDNA (pLV[Exp]-Puro-EF1A>hTXNIP[NM_006472.6]) plasmids were obtained from VectorBuilder (Neu-Isenburg, Germany). Briefly, 10 µg of either plasmid, along with lentiviral pAX (5 µg) and VSV-G (2.5 µg), were transfected into ~3 × 10⁶ 293 T cells on a 100 mm plate in 7 mL DMEM (10% FBS, 4 mM L-glutamine, 1% pen-strep) using 9.5 mM CaCl₂ and HBS buffer at pH 7.05. Following overnight incubation, the media was refreshed, and lentivirus-containing supernatants were collected after 30 h, concentrated by overnight centrifugation and stored at −80 °C. LNCaP or PC-3 cells were seeded at 6.5 × 10⁵ cells/T25 flask and infected at a MOI of 2. After 48 h, 1 µg/mL puromycin was added for one week to select stably transduced cells. TXNIP overexpression was confirmed by immunoblotting and qPCR.

### Animal models and procedures

Transgenic adenocarcinoma of the mouse prostate [C57BL/6-Tg (TRAMP)8247Ng] [18] and mice bearing floxed *Txnip* gene (*Txnip*^Flox^; B6;129-*Txnip*^tm1Rlee^/J) [19] were purchased from The Jackson Laboratory (Bar Harbor, ME, USA). *Txnip*^Flox^ were crossed with Cre Mice to obtain a *Txnip*^−/−^ offspring. Homozygous *Txnip*^−/−^ were then crossed with TRAMP mice, and experimental TRAMP-*Txnip*^−/−^ were obtained by backcrossing to F3. Mice were bred and maintained under standard conditions in the Animal Facility of the University of Oviedo, adhering to the guidelines of the Ethics Committee for Animal Experimentation (PROAE 01/2020, PROAE 17/2023, and PROAE 20/2021) in compliance with Directive 2012/63/EU. ADT model in mice was established by performing bilateral orchiectomy on males at 18 weeks of age. Sham-operated mice underwent the same surgical procedure without testis removal. Animals were sacrificed at 24 weeks of age. Glycemia was assessed using a standard glucometer. Circulating testosterone levels were measured in plasma samples with Testosterone ELISA kit (Cayman Chemical, Ann Harbor, MI, USA) following manufacturer’s instructions. Prostate tissues were collected for molecular biology (snap-frozen) and histological analysis (PFA 4%) using standard paraffin embedding protocols.

### Patient samples

All studies involving human samples were conducted in accordance with the Declaration of Helsinki. Patient samples and data used in this study were provided by the Biobank of the Principality of Asturias (BioPA) (National Registry of Biobanks B.0000827, PT23/00077 funded by ISCIII and co-funded by the European Union), integrated within the ISCIII Platform for Biomodels and Biobanks. Samples were processed following standard operating procedures and with the appropriate approval of the Ethics and Scientific Committees (CEImPA 2022.073). Prostatectomy samples were collected from Hospital Universitario Central de Asturias (*n* = 15) and Hospital Valle del Nalón (*n* = 6).

Experimental groups were defined as follows: (1) Normal versus tumoral TXNIP levels: samples from radical prostatectomy patients who had received no pharmacological treatment (*n* = 15) were used. (2) Prediction of ADT response based on TXNIP levels: samples from patients who underwent ADT treatment with available follow-up clinical data (*n* = 16) were analyzed. (3) TXNIP levels pre- and post-ADT: prostatectomy samples were collected from prostate cancer patients post-ADT treatment, including both prostate tumors and metastasis sites (*n* = 16).

Histological diagnoses were confirmed by a pathologist, and all samples were processed according to standard protocols for formalin-fixed paraffin-embedded samples at the Pathological Anatomy Service of Hospital Universitario Central de Asturias. To evaluate TXNIP levels for ADT response prediction, cohorts from the two hospitals were analyzed separately. General patient data are provided in Table SII.

### Protein Extraction and Immunoblotting

For detailed protocol see Supplementary material. Cells were seeded at an initial density of 25,000 cells/mL. At the designated experimental time points, cells were lysed with Tri-detergent buffer supplemented with Complete™ protease inhibitor cocktail and phosphatase inhibitors. After 30 min on ice, lysates were centrifuged, and the supernatant was collected.

For nuclear protein extraction, a modified protocol from Go and Miller (1992) was used. Briefly, cells were collected and incubated with Buffer A on ice for 5 min. Cells were lysed in Buffer A containing 0.2% NP-40 for 10 min. Nuclei were isolated by centrifugation, resuspended in Buffer B and incubated on ice for 15 min. Buffer C was added and nuclear proteins were collected by centrifugation. Protein concentration was determined using the Bradford assay.

Protein extracts were resolved by SDS-PAGE and transferred to PVDF membranes using the Trans-Blot Turbo Transfer System (Bio-Rad, Hercules, CA, USA), following the manufacturer’s instructions. Membranes were blocked in 5% non-fat dry milk for chemiluminescence detection or Li-Cor blocking buffer for fluorescence detection. Antibodies and dilutions are detailed in Table SIII. Imaging was performed on an Odyssey XF (Li-Cor, Lincoln, NE, USA) for both chemiluminescence and fluorescence.

### Immunostaining

For detailed protocol see Supplementary material. LNCaP cells were fixed with 4% PFA. After washing with PBS, cells were blocked and permeabilized (0.5% BSA, 0.1% Tween TBS) for 30 min. Primary antibodies were incubated overnight at 4 °C followed by secondary antibody. Dilutions are specified in Table SIII.

For tissue immunofluorescence (IF), 5 µm sections were deparaffined and rehydrated. Antigen retrieval, blocking and permeabilization steps for the different IF are detailed in Table SIV. Primary antibody dilutions are provided in Table SIII. KI67 IHC was performed automatically using the Roche Discovery ULTRA system.

### RNA extraction and RT-qPCR

For detailed protocol see Supplementary material. Cells were seeded at 25 000 cell/mL. After the given experimental times and treatments, cells were collected in NZYol. For prostate tissue samples, pieces of approximately 50 mg were homogenized in 1 mL of NZYol.

RNA was purified following the manufacturer’s instructions. RNA (1 µg) was used for first-strand cDNA synthesis, and qPCR was performed using SYBR Green-based probes in a Quant Studio 5 thermocycler (Thermo Fisher, Waltham, MA, USA). Primer sequences are detailed in Table SV. Relative expression levels were calculated (^ΔΔ^Ct method).

### Chromatin immunoprecipitation

LNCaP cells were seeded in 150 mm plates and harvested at 70% confluency. The assay was conducted with Simple ChIP (Magnetic beads) kit (Table SI) following manufacturer’s instructions. Briefly, chromatin-protein complexes were crosslinked with PFA. Chromatin was digested and then precipitated overnight with 1:100 anti-AR antibody (Table SI). Samples were incubated with magnetic G protein beads, AR was eluted, and the DNA sequence was purified by column purification included in the kit. To confirm the union of AR to the *TXNIP* promoter, the sequence was amplified by PCR, and DNA enrichment was calculated by qPCR amplification compared to input (non-immunoprecipitated) samples. Primers used can be found in Table SV.

### Seahorse real-time metabolic cell analyzer

Seahorse^TM^ XF HS Mini Analyzer was used to assess glycolysis and mitochondrial respiration. For both tests, 25,000 cells/well were seeded in a poly-lysin coated Seahorse plate and maintained according to manufacturer’s instructions. Glycolytic Rate Assay and Mito Stress Test were analyzed using Agilent Cell Analysis tools.

### Extracellular L-lactate measurement

Cells were plated at 25 000 cell/mL. After 2 days, the media was collected, centrifuged at 4 °C, and transferred to clean tubes kept on ice. L-lactate levels were measured using a colorimetric assay following manufacturer’s instructions (Sigma, Rahway, NJ, USA). DNA content in each well, assessed using Hoechst assay, was used to normalize cell quantities.

### Mitochondrial membrane potential (MMP) assessment

Cells were seeded at 25,000 cells/mL. After 2 days, cells were collected by trypsinization, resuspended in JC-1 dye (2 µM) in Hank’s balanced solution, and incubated for 20 min at 37 °C. JC-1 fluoresces green in the cytoplasm and forms red-fluorescent J-aggregates in the mitochondria based on membrane potential. Fluorescence was measured using a Cytoflex S cytometer (Beckman Coulter, Brea, CA, USA), and the 590 nm (red)/529 nm (green) fluorescence ratio was calculated. Experiments were performed in triplicate, with at least 10 000 events analyzed per replicate. Data analysis was conducted using FlowJo software (BD, v10.9.0).

### Metabolic flux analysis

Cells were seeded at 25,000 cells/mL. RPMI medium supplemented with 2 g/L U-13C-glucose was added and incubated overnight. Intracellular metabolites were extracted using a double extraction with 100% methanol, followed by a single extraction with milli-Q water [20]. Derivatization was performed as previously described [21]. Gas chromatography-mass spectrometry analysis was performed using a 7890 A system with an autosampler 7693 A coupled to a triple quadrupole mass spectrometer 7000 Series GC/MS Triple Quad (Agilent Technologies, Santa Clara, CA, USA). The equilibration, injection and detection conditions are detailed in Supplementary Methods. The relative contribution of isotope patterns in the experimental mass spectra was calculated via multiple linear regression. Enrichment and pathway analyses were performed using MetaboAnalyst 6.0 [22].

### In vitro redox species detection by fluorescent probes

Cells were seeded at 25,000 cells/mL. After 2 days, cells were trypsinized and resuspended in Hank’s balanced solution containing the following probes: DCFH-DA (50 nM), DHE (500 nM), and MitoSOX (1 µM) with MitoGreen (20 nM) for measuring mitochondrial superoxide production normalized to mitochondrial mass. Probes were incubated for 20 min at 37 °C, centrifuged, and resuspended in Hank’s solution for fluorescence acquisition using a Cytoflex S cytometer (Beckman Coulter). Three replicates per condition and at least 10,000 events per replicate were analyzed. Data analysis was performed using FlowJo software (BD, v10.9.0). The experiment was repeated three times.

### Proliferation and cell cycle analysis

For detailed protocol see Supplementary material. Cells were seeded at 25,000 cells/mL and allowed to attach for 48 h. Each experiment was performed in triplicate and repeated three times.

For cell cycle analysis, fixed cells were resuspended at 10^6^ cells/mL in a propidium iodide solution (PI) (PI 100 µg/mL + RNase A 100 µg/mL + glucose 1 g/L). PI fluorescence was measured using a Cytoflex S cytometer, 10,000 events per replicate analyzed. Data analysis was performed using FlowJo software (BD, v10.9.0).

### Apoptosis assay

For detailed protocol see Supplementary material. Cells were plated at 25,000 cells/mL. After treatments, cells were resuspended in a working solution of 150 nM YOPRO-1® + 500 nM PI. Fluorescence was measured using a Cytoflex S cytometer. Three replicates per condition and 10,000 events per replicate were analyzed. Experiments were repeated three times, and data analysis was performed using FlowJo software (BD, v10.9.0).

### Spheroid formation

Cells were plated at 25,000 cells/mL in a round-bottom ultra-low adhesion 96-well plate. Spheroids were allowed to form for 10 days, with medium changed every 3 days. Bright-field images were captured; image analysis methods are described in Image Analysis section.

### Invasiveness assay by colony formation in Matrigel

LNCaP cells at a density of 100 cells/μL were resuspended in a drop of 10 μL of 70% Matrigel-RPMI in a 96 well plate. After solidification, complete RPMI was added. Colonies were left for 10 days. Bright-field images were captured; image analysis methods are described in Image Analysis section.

### MEFs extraction and culture

Mouse embryos (E12.5) from *Txnip*^WT^ and *Txnip*^−/−^ mice were collected, cleaned, and minced in PBS under sterile conditions. Tissue was disaggregated with 0.25% trypsin-EDTA for 30–60 min, neutralized with complete medium, and plated for attachment in 60 mm plates. At confluence, cells were passaged using 0.05% trypsin-EDTA. MEFs were cultured in DMEM high-glucose medium supplemented with 10% FBS, 2 mM L-glutamine, and 1% pen-strep. Growth assessment followed the cell proliferation protocol. Senescence was evaluated using a β-galactosidase kit (Cell Signaling, Danvers, MA, USA) according to the manufacturer’s instructions.

### Organoid cell culture

Organoids from TRAMP-*Txnip*^WT^ and TRAMP-*Txnip*^−/−^ mice were established as previously described [23]. Prostate tissue from 18-week-old mice was excised, mechanically minced, and digested with 5 mg/mL collagenase type II and 10 µM *anoikis* inhibitor Y-27632 for 30 min. A single-cell suspension was obtained using TrypLE™ with 10 µM Y-27632 for 10 min. 20,000 cells were seeded in 40 µL Growth Factor Reduced Matrigel drops in 24-well plates. Medium composition is detailed in Table SVI. The expansion medium was changed every 3 days. Ten organoid lines (up to passage 10) were established.

### Organoid viability assay

Cells were seeded at a density of 250 viable cells in a 9 μL drop of 70% Matrigel in a 96-well plate. After 3 days, expansion medium with or without dihydrotestosterone (DHT) was added. After 5 days, viability was measured using CellTiter-Glo^TM^ kit according to manufacturer’s instructions. Six replicates per primary cell line and per experimental condition were performed. Viability percentage was calculated vs CONTROL (+DHT).

### Organoid imaging

Organoids were released from the Matrigel, incubating each well with 500 μL 2 mg/mL dispase for 45 min at 37 °C. Organoids were transferred to a microcentrifuge tube and washed once with ice-cold PBS. The organoids were centrifuged at 250 × *g* and fixed in 4% PFA (overnight, 4 °C). Next day, after removing the PFA, the organoids were embedded in agarose-based medium HistoGel^TM^. Once solidified, the agarose drop was transferred to a conventional histology cassette processed following standard procedures for dehydration and paraffin embedding.

### Image analysis

#### Spheroid area and solidity

Bright-field images were captured at 10x magnification using an EVOS™ XL Core Imaging microscope (Invitrogen, Waltham, MA, USA). Analysis was performed using FIJI software. Images were converted to 8-bit, and the “Max Entropy” threshold was applied to detect the spheroid. The images were then converted to binary format. The “Analyze Particles” function was used with the following settings: (a) Minimum size: 5000 px; (b) circularity range: 0.20–1.00.

A region of interest (ROI) was created to measure the “area” and “solidity” in the original image.

#### 3D colonies count

Bright-field images were taken at 10x magnification with an EVOS^TM^ XL Core Imaging microscope (Invitrogen). Threshold method was run on the 8-bit images. A mask was created, and used for analyzing particles (min size 350, no max size) to obtain the number of colonies detected.

Analysis was performed using FIJI software. Images were converted to 8-bit, and the “Percentile” threshold was applied. The “Analyze Particles” function was used in the resulting mask with the following settings: a) Minimum size: 350 px (no max size).

#### KI67 positive detection in epithelia

KI67 images stained with DAB were scanned with a NanoZoomer SQ Hamamatsu scanner at 40x magnification. Image analysis was performed using QuPath software [24]. The images were set to the ‘BrightField_H_DAB’ vector. The Positive Cell Detection tool was run to identify cells using the Hematoxylin algorithm in place at a resolution of 0.5 μm/px. The following settings were applied: (a) Minimum-Maximum Area: 7.0–500 μm; (b) Cell detection threshold: 0.07; and (c) Positive detection threshold (DAB nucleus): 0.2. The “Object Classifier” tool was trained to differentiate epithelia and stroma, and percentage of positive detection in each tissue was calculated.

#### Fluorescence intensity in tissue

For epifluorescence, all images were taken with Nikon 80i microscope at same exposure, gain (1x), and binning (1 × 1). For confocal microscopy images were taken with a Leica Sp8 microscope with the same pinhole (1.00), laser intensity, and gain. Image analysis was performed using QuPath software. “Cell Detection” tool was used to detect cells with the blue channel (DAPI) at a resolution of 0.5 μm px and the following parameters: (a) Minimum-Maximum Area: 40–400 μm; (b) Cell Detection threshold: 16.0, and (c) Cell Expansion: 5.0 μm. The “Object Classifier” tool was trained to classify detections into tumoral or stromal. Mean fluorescence of each image was calculated, and the mean of images for the same sample was used to perform the statistical analysis.

### Statistics

Unless specified, all experiments were repeated three independent times, and results are expressed as mean ± SEM. Normality was assessed with the Shapiro–Wilk test (*n* = 3) or the Kolmogorov–Smirnov test for larger samples. For comparisons between two groups, Student’s *t*-test or the Mann–Whitney test was used based on normality. Multiple comparisons were analyzed with one-way ANOVA followed by Tukey’ test. Proportions -were compared using the *χ*^2^ test. Statistical significance was set at *p* < 0.05. Data was analyzed and visualized using GraphPad Prism 8.

## Results

### TXNIP is downregulated in PCa, and its expression depends on androgen signaling

**Fig. 1:**
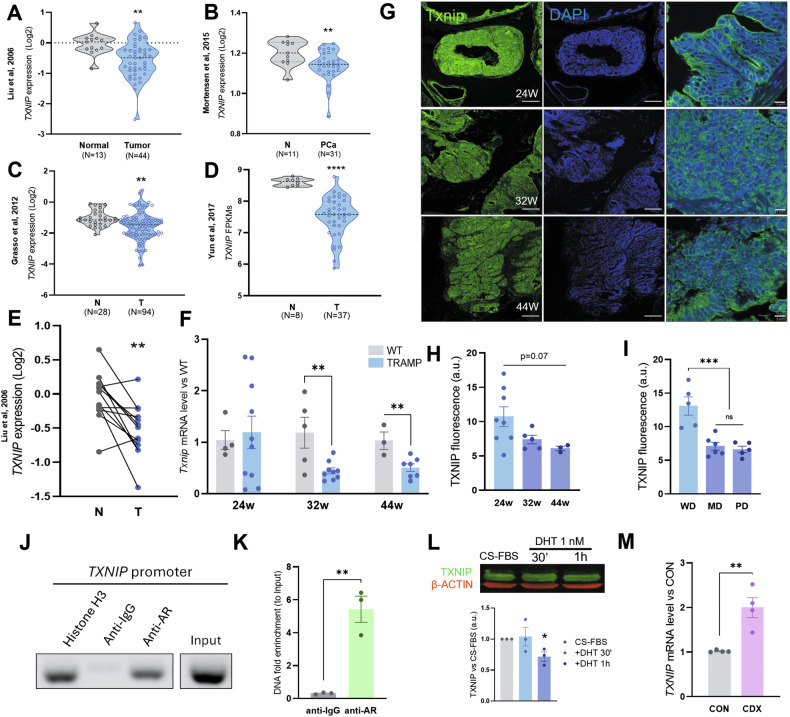
TXNIP is downregulated in PCa and its expression depends on androgen signaling. **A** Relative *TXNIP* mRNA expression in normal and tumoral tissue obtained from PCa patients, data taken from Liu et al. [25] open database. **B** *TXNIP* mRNA expression in normal and tumoral tissue obtained from PCa patients, data taken from Mortensen et al. [26] open database. **C** *TXNIP* mRNA expression in normal and tumoral tissue obtained from PCa patients, data taken from Grasso et al. [28] open database. **D** *TXNIP* mRNA expression shown as FPKMs in normal, PCa and CRPC tissue of patients from the public data generated by Yun et al. [27]. **E** Comparison of *TXNIP* mRNA expression in normal and tumoral tissue of the same patients from data available in Liu et al. [25]. Dots matched represent the same patient (two-tailed paired T-test). **F** *Txnip* mRNA expression in a transgenic mouse model of prostate cancer; wild-type (WT) or tumoral (TRAMP) prostate tissue was compared at 24, 32, and 44 weeks of age. **G** Representative images of Txnip immunofluorescence (green) in prostate tissue from TRAMP mice at 24, 32, and 44 weeks-old. DAPI was used as countersatining (blue) 20x magnification, scale bar 100 μm. Right column shows the TXNIP-DAPI merge at 630x, scale bar 10 μm for more detail. **H** Txnip protein levels in tumoral prostate tissue of TRAMP mice at 24, 32, and 44 weeks-old. **I** Txnip protein levels in tumoral prostate tissue of TRAMP mice with samples grouped by tumor diagnosis. WD well differentitated, MD moderately differentiated, PD poorly differentiated. **J** PCR products of *TXNIP* promoter from LNCaP cells chromatin precipitated with antibodies anti-histone H3 for positive control, anti-IgG, and anti-AR. Amplification is observed in chromatin purified from anti-AR, but not from anti-IgG. 2% of non-precipitated chromatin was used as input sample. **K** DNA fold enrichment of *TXNIP* promoter vs input samples after immunoprecipitation in LNCaP cells. **L** Immunoblotting of TXNIP protein from LNCaP cells cultured in CS-FBS conditions and 1 nM DHT added at indicated times. Normalization to CS-FBS (w/o DHT) of TXNIP/ACTIN ratio was performed for the three independent replicates. **M** *TXNIP* relative mRNA expression in LNCaP cells incubated with the antiandrogen Casodex (CDX) compared to untreated (CON) cells. Data represented as mean ± SEM of four different experiments. N of all experiments is indicated in their respective figures. *p-value < 0.05; ** *p*-value < 0.01; ***p value < 0.001; ****p-value < 0.0001. *T*-test for **A**, **C**–**F**, **K**, **M**. Two-tailed unpaired Mann–Whitney test for B. One-way ANOVA test followed by Tukey’s multiple comparisons test for (**H**, **I**).

The expression of *TXNIP* is reduced in PCa compared with normal tissue, which was reported by Guo et al. [17] based on the TCGA dataset. We confirmed these results in four additional PCa cohorts [25–28]: Liu (*p* = 0.0034, Fig. 1A), Mortensen (*p* = 0.0086, Fig. 1B), Grasso (*p* = 0.009, Fig. 1C), and Yun (*p* < 0.0001, Fig. 1D). The levels were also lower when normal and tumor tissue were compared within the same patient (Liu; Fig. 1E, *p* = 0.0017). Further details on clinical data from the patients enrolled in the cohorts can be found in Table SVII.

Consistent with these observations, the TRAMP PCa mouse model showed a reduction in TXNIP at both RNA and protein level during tumor progression when compared with their WT counterparts at 32 and 44 weeks (Figs. 1F–I, and S1A). This downregulation is also observed in prostate cell lines (Fig. S1B). *TXNIP* has been proposed to be regulated by androgens in other models [29]. Using PROMO 3.0 Software [30], we identified androgen response elements (AREs) within human *TXNIP* sequence. To confirm the actual binding of AR to *TXNIP* DNA sequence, we performed a ChIP assay with an anti-AR antibody in LNCaP cells. Interestingly, DNA purified from precipitated AR was 6-fold enriched in the sequence of *TXNIP* promoter, unlike DNA purified from an IgG antibody (Fig. 1J, K). Androgen-dependent human LNCaP cells showed a significant decrease in TXNIP expression after 1 h of treatment with Di-Hydro-testosterone (DHT) (*p* = 0.02) (Fig. 1L). Conversely, there is a significant increase in *TXNIP* levels when LNCaP cells are cultured in androgen blockade conditions using either 20 μM Casodex® (Bicalutamide) as a model for ADT (*p* < 0.005; Fig. 1M) or CS-FBS (*p* < 0.005, Fig. S1B). Furthermore, the addition of 1 nM DHT prevented the *TXNIP* rise (*p* < 0.01, Fig. S1C). Thus, we can conclude TXNIP expression is directly regulated by androgens in prostate tissue.

### TXNIP induces metabolic reprogramming by inhibiting glycolysis in androgen-dependent PCa cells

**Fig. 2:**
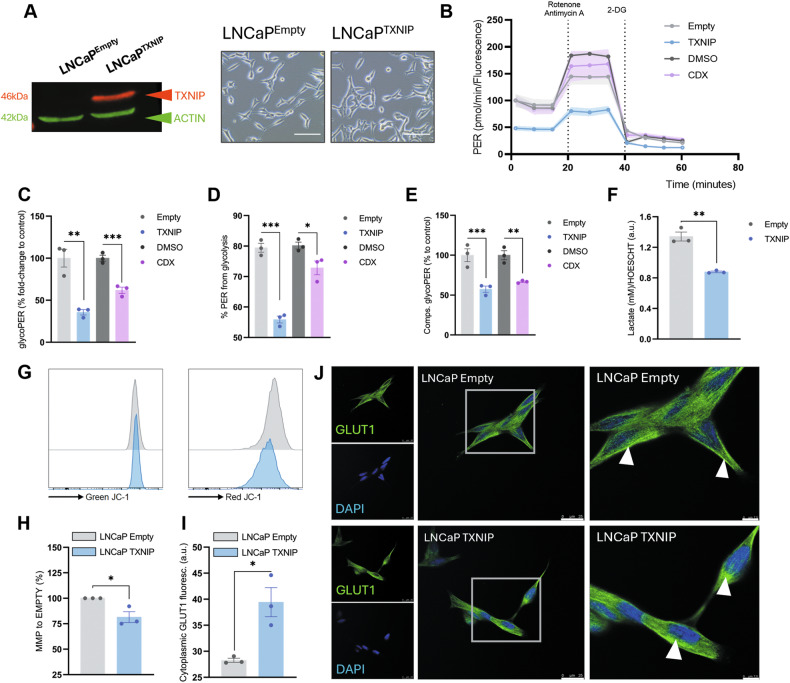
TXNIP induces metabolic reprogramming by inhibiting glycolysis in androgen-dependent PCa cells. **A** Representative immunoblotting showing TXNIP overexpression in transfected LNCaP TXNIP cells compared to control LNCaP Empty cells. On the right, representative bright-field micrographs of LNCaP Empty and LNCaP TXNIP cell lines. 20x magnification, scale bar 50 μm. **B** Proton Efflux Rate (PER) was measured following sequential injection of Rotenone/Animycin A and 2-deoxyglucose (2-DG) as indicated. **C** Glycolysis-derived Proton Efflux Rate (glycoPER) in basal conditions of LNCaP Empty and LNCaP TXNIP cells and in LNCaP DMSO and LNCaP 20 μM CDX (ADT). **D** Percentage of protons released due to glycolysis that represent the measured PER of LNCaP Empty, LNCaP TXNIP, LNCaP DMSO and LNCaP 20 μM CDX cells. **E** GlycoPER from compensatory glycolysis, after cells (LNCaP Empty, LNCaP TXNIP, LNCaP DMSO, and LNCaP 20 μM CDX) are challenged with Rotenone/Antimycin (**A**). **B**, **C** LNCaP TXNIP normalized to LNCaP Empty, and LNCaP CDX normalized to LNCaP DMSO. *N* = 3 from a representative experiment of three biological replicates. **F** Extracellular lactate measured in the culture media of both cell lines. **B**–**F** measures are normalized against number of cells by DNA-content measurement with Hoescht. *N* = 3. Data represented as mean ± SEM of a representative experiment from three different experiments. **G** Histograms of red/green fluorescence determined by JC1 probe measurement to assess mitochondrial membrane potential (MMP). **H** Ratio red/green was normalized versus Empty group in each of the three different experiments, with *N* = 3 replicates. Data as mean ± SEM of three different experiments. **I** Cytoplasmic GLUT1 determination in LNCaP Empty and LNCaP TXNIP. *N* = 3. **J** representative images of GLUT1 staining (GLUT1 in green, DAPI in blue). Left and centre panels 63x magnification, scale bar 25 μm. Right panels show in detail the area marked in central panels. White arrows point the main accumulations of GLUT1 in the cells. 150x magnification, scale bar 7.5 μm. For **C**–**I** unpaired *T*-test was performed *p-value < 0.05; **p-value < 0.01; ***p-value < 0.001; ****p-value < 0.0001.

To study the impact of TXNIP in PCa cells, we overexpressed TXNIP in LNCaP cells with the human cDNA (LNCaP TXNIP) (Figs. 2A and S2A, B).

As mentioned above, TXNIP plays a pivotal role in glucose metabolism, and, therefore, we characterized glycolytic activity in TXNIP-overexpressing cells, as well as in CDX-treated LNCaP cells, given that anti-androgens recover the levels of this protein. Basal proton efflux rate (PER) derived from glycolysis was significantly reduced in both LNCaP TXNIP and LNCaP CDX compared to cells transduced with an empty vector (LNCaP Empty) or incubated with DMSO (*p* < 0.01 and *p* < 0.001, respectively; Fig. 2B, C).

The percentage of PER from glycolysis decreased from 79.4% in LNCaP Empty to 55% in LNCaP TXNIP (*p* < 0.001; Fig. 2D), as well as from 80.2% in LNCaP DMSO to 72.9% in LNCaP CDX (*p* < 0.05, Fig. 2D). Additionally, compensatory glycolysis, reflecting the cellular capacity to meet energy demands under stress, was reduced by half in LNCaP TXNIP and in LNCaP CDX cells compared to their respective controls (*p* < 0.01 and *p* < 0.05 respectively; Fig. 2E). These findings place TXNIP overexpression and anti-androgen CDX treatment on a comparable metabolic level, suggesting that the restoration of TXNIP levels may represent a key mechanism through which anti-androgens exert part of their metabolic effects in LNCaP cells. Finally, in line with these findings, LNCaP TXNIP cells released approximately 35% less lactate to the extracellular medium compared to LNCaP Empty cells (*p* < 0.01; Fig. 2F). The strong decrease in glycolysis and lactate concurs with a drop in mitochondrial membrane potential, as assessed with the JC-1 probe (*p* < 0.05; Fig. 2G, H), and in their capacity to reduce MTT reagent (Fig. S2C). As mentioned above, TXNIP sequesters GLUT1 in the cytoplasm, therefore, we studied the subcellular location of this glucose transporter in LNCaP cells. As expected, GLUT1 was found mainly in the cytosolic compartment in LNCaP TXNIP cells compared to LNCaP Empty (Fig. 2I, J). This GLUT1 subcellular location would explain the decrease in glucose uptake and the subsequent shift in metabolic profile in TXNIP-overexpressing cells.

Furthermore, we determined a change towards aerobic metabolism by a MitoStress Seahorse^TM^ assay (Fig. S2D, E). Measured ATP-production coupled respiration confirmed the switch to respiratory metabolism in LNCaP TXNIP cells (*p* < 0.05; Fig. S2D, F). Trends towards higher basal respiration (Fig. S2D, G) and maximal respiration (Fig. S2D, H) were observed, but these did not reach statistical significance (*p* = 0.1 and *p* = 0.09, respectively). Finally, total mitochondrial biomass remained unchanged upon TXNIP overexpression (Fig. S2I).

**Fig. 3:**
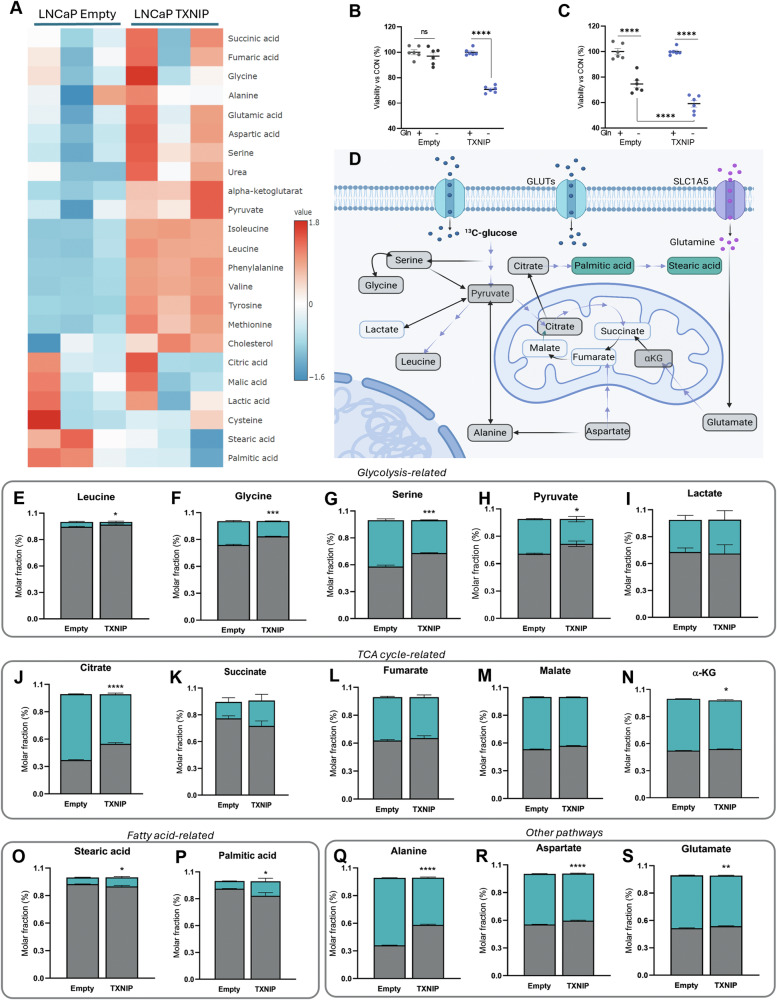
Metabolomic profile of TXNIP overexpressing cells. **A** Heatmap showing the relative concentration of detected metabolites in TXNIP overexpressing LNCaP cells compared to control LNCaP Empty cells (3 left columns LNCaP Empty, 3 right columns LNCaP TXNIP). Higher concentrations are depicted in red tones, while lower concentrations are in blue tones. Cell viability of LNCaP Empty and LNCaP TXNIP after being grown in absence or presence of glutamine (Gln) for 48 h **B** and 72 h **C**. Data shown as mean ± SEM of percentage of viability compared to control (+Gln) of one representative experiment from three independent replicates. **D** Schematic diagram of the metabolic flux analysis (MFA) in the cell and metabolites detected for 13C enrichment. Metabolites in gray are non-enriched in 13C in LNCaP TXNIP; metabolites in dark greenish blue are enriched in 13C in LNCaP TXNIP; metabolites in light blue did not show differences in 13C enrichment between LNCap Empty and LNCaP TXNIP. **E–S** Percentage of molar fraction of metabolites enriched (dark greenish blue) or non-enriched (gray) in 13C. For simplicity, metabolites are grouped in direct or indirect relation to different metabolic pathways: Glycolysis-related: **E** Leucine; **F** Glycine; **G** Serine; **H** Pyruvate; **I** Lactate. TCA cycle-related: **J** Citrate; **K** Succinate; **L** Fumarate; **M** Malate; **N** α-ketoglutarate. Fatty-acid synthesis-related: **O** Stearic acid; **P** Palmitic acid. Others: **Q** Alanine; **R** Aspartate; **S** Glutamate. Data shown as mean ± SD of three technical replicates. For **B** to **S** unpaired T-test was performed *p-value < 0.05; **p-value < 0.01; ***p-value < 0.001; ****p-value < 0.0001.

As TXNIP inhibits the antioxidant protein TRX, we also investigated redox metabolism in TXNIP-overexpressing cells. No significant differences were observed in basal levels of reactive oxygen species (ROS) in the cytoplasm (Fig. S3A), superoxide ion (O₂•⁻) levels in the cytoplasm (Fig. 3SB), or mitochondria (Fig. S3C).

Total antioxidant protein levels, including SOD1, SOD2, TRX1, TRX2, catalase, and PRDX6, were similar between groups (Fig. S3D–K). However, GPX4, a specific scavenger for lipid peroxidation, showed a consistent 25% increase in LNCaP TXNIP cells (*p* < 0.001; Fig. S3I). Thus, high levels of TXNIP in LNCaP cells induces a metabolic switch to a less glycolytic and more oxidative metabolism mediated by GLUT1 internalization, not causing major changes in redox state.

### Metabolic reprogramming in TXNIP-overexpressing cells

To characterize the metabolic changes induced by TXNIP, we performed a metabolomic assay. Cells overexpressing TXNIP showed a significant increase in amino acids (methionine, tyrosine, valine, phenylalanine, leucine, serine) and urea (Fig. 3A). Furthermore, α-ketoglutarate (α-KG) was the most increased metabolite in LNCaP TXNIP compared to LNCaP Empty (Fig. 3A). On the contrary, fatty acids like palmitic acid and stearic acid were decreased in LNCaP TXNIP (Fig. 3A).

Given the increase in nitrogen-containing products, and especially in α-KG, an intermediate from glutamine to feed the TCA cycle, and considering that glutamine is a key nutrient for cancer cells, we checked whether this amino acid may compensate for the absence of glucose in LNCaP TXNIP cells. Under glutamine-deprived conditions, LNCaP TXNIP cells showed a marked reduction in viability compared to LNCaP Empty cells at 48 h (*p* < 0.0001; Fig. 3B) and 72 h (*p* < 0.0001; Fig. 3C).

Next, we investigated the use of glucose in both cell lines by metabolic flux analysis (MFA). Measured metabolites and changes in their 13C enrichment are depicted in Fig. 3D. Expectedly, glycolysis-related metabolites including leucine (Fig. 3E*p* < 0.05), glycine (Fig. 3F*p* < 0.0001), serine (Fig. 3G*p* < 0.01) and pyruvate (Fig. 3H*p* < 0.05) were less enriched in 13C coming from glucose in LNCaP TXNIP cells, while intracellular lactate enrichment remained unchanged (Fig. 3I). Citrate, a key TCA cycle intermediate, was less enriched in LNCaP TXNIP cells (*p* < 0.0001; Fig. 3J). However, downstream TCA metabolites (succinate, fumarate, and malate) showed no significant changes (Fig. 3K–M). α-KG again showed significantly less 13C enrichment (Fig. 3N*p* < 0.05). Noteworthily, fatty acids such as stearic acid (Fig. 3O*p* < 0.05) and palmitic acid (Fig. 3P*p* < 0.05) exhibited higher 13C enrichment in LNCaP TXNIP cells.

Finally, amino acids such as alanine (Fig. 3Q*p* < 0.001), aspartate (Fig. 3R*p* < 0.001), and glutamate (Fig. 3S*p* < 0.01) were less enriched in LNCaP TXNIP cells, supporting the hypothesis that these cells rely on glutamine-derived pathways rather than glycolysis for amino acid biosynthesis.

### TXNIP induces proliferation arrest in PCa androgen-dependent cells

**Fig. 4:**
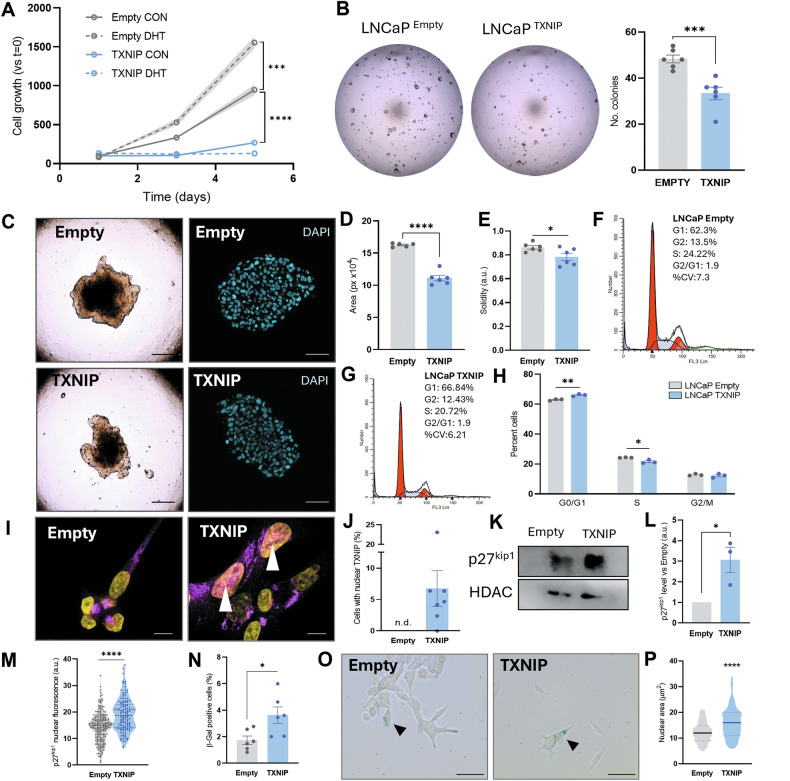
TXNIP induces proliferation arrest in PCa androgen-dependent cells. **A** Cell growth rate of LNCaP Empty and LNCaP TXNIP with or without 5 nM DHT. *N* = 6. **B** Colony formation assay in 3D conditions. Representative images of colonies formed by LNCaP Empty and LNCaP TXNIP in Matrigel. On the right, quantification of a representative experiment of the number of colonies counted from both cell lines. **C** Bright-field (left) and DAPI staining (right) images of 3D spheroids formed by LNCaP Empty and LNCaP TXNIP cells. BF magnification 10x, scale bar 100 μm; DAPI 20x, scale bar 25 μm. Quantifications of area, **D**, and solidity, **E**, of the spheroids. *N* = 6 from a representative experiment. Representative graphs showing the distribution of LNCaP Empty, (**F**), and LNCaP TXNIP, (**G**) cells in the different phases of the cell cycle (G1, S, G2). **H** Quantification of the percentage of LNCaP Empty and LNCaP TXNIP cells in the different phases of the cell cycle (G1, S, G2). **I** Representative images of TXNIP immunostaining (purple) in LNCaP Empty and TXNIP cells (DAPI in yellow as counterstaining). White arrows show TXNIP accumulation in the nucleus of LNCaP TXNIP cells. 150x magnification, scale bar 7.5 μm. **J** Percentage of cells from both groups presenting nuclear localization of TXNIP. **K** Representative immunoblotting of p27kip1 from nuclear extracts of LNCaP Empty and LNCaP TXNIP cells. HDAC is shown as nuclear housekeeping. **L** Quantification of p27kip1 levels from the three independent experiments. **M** Quantification of p27kip1 nuclear fluorescence assessed by IF in LNCaP Empty (*N* = 211 cells) and LNCaP TXNIP (*N* = 132 cells) cells. **N** Quantification of positive cells for β-galactosidase staining represented as percentage of the total. **O** Representative images of β-galactosidase staining to assess senescence in LNCaP Empty and LNCaP TXNIP. Arrowheads show positive cells. 40x magnification, scale bar 15 μm. **P** Quantification of the nuclear area in μm^2^ of LNCaP Empty and LNCaP TXNIP cells (Empty *N* = 175 and TXNIP *N* = 165 cells analyzed from a representative experiment from three independent experiments). For **A**, **B**, **D**, **E**, **H**, **L**, **N** and **P** unpaired *T*-test was performed. For **M** Mann–Whitney test was performed. Unless specified otherwise, data was represented as mean ± SEM of three independent experiments. *p-value < 0.05; **p-value < 0.01; ***p-value < 0.001; ****p-value < 0.0001.

Next, we investigated whether TXNIP recovery influence other key aspects of prostate tumor cell biology. LNCaP TXNIP cells had a significantly lower growth rate (doubling time LNCaP Empty 42 h vs LNCaP TXNIP 54 h, *p* < 0.0001; Fig. 4A). Also, TXNIP overexpressing cells are less sensitive to growth-promoting effect of DHT (Fig. 4A).

We also examined the invasiveness and growth of LNCaP TXNIP cells in 3D culture. A Matrigel colony formation assay showed a reduced invasive capacity in LNCaP TXNIP cells (*p* < 0.001; Fig. 4B). Furthermore, 3D spheroids formed by LNCaP TXNIP had reduced area (*p* < 0.0001; Fig. 4C, D) and solidity (*p* < 0.05; Fig. 4C, E).

These differences were not seen in androgen-independent PC-3 cell line overexpressing TXNIP (Fig. S4A, B). No changes were seen in 2D growth (Fig. S4C), viability (Fig. S4D), or 2D colony formation (Fig. S4E, F) between PC-3 Empty and PC-3 TXNIP cells, suggesting that TXNIP’s effects are specific to androgen-dependent PCa cells.

We next sought to identify the mechanisms underlying the slower growth rate observed in LNCaP TXNIP cells. No changes were observed in apoptosis (Fig. S4G–I). However, approximately 5% of LNCaP TXNIP cells were arrested in the G1 phase (*p* < 0.05), with the correspondent decrease in the S phase population (*p* < 0.01; Fig. 4F–H).

TXNIP is primarily localized in the cytoplasm (Uniprot Ref Q9H3M7), but it can translocate to the nucleus or mitochondria under certain conditions [11, 31]. LNCaP TXNIP cells exhibited higher percentage of cells with nuclear TXNIP (Fig. 4J) and a three-fold increase of nuclear p27^kip1^, a critical regulator of the G1 phase through CDK inhibition assessed by western-blot (*p* < 0.05; Fig. 4K, L) and IF (*p* < 0.0001; Figs. 4M and S4J). To confirm the role of TXNIP in p27^kip1^ accumulation, we treated LNCaP with 30 μM SRI-37330, a selective inhibitor of TXNIP. At 48 h, a strong diminution of p27^kip1^ was observed (Fig. S4K). Other regulators of the cell cycle (p-Rb and Cyclin A) showed no significant differences (Fig. S4L). Finally, given the less proliferative metabolism and arrest in cell cycle, we also assessed senescence in LNCaP TXNIP. We identified a significant increase in senescent cells assessed by β-galactosidase staining in LNCaP TXNIP cells (Fig. 4N–O; *p* < 0.05), and confirmed by significantly increased nuclei area, another common feature of senescent cells (Fig. 4P; *p* < 0.0001). These results altogether show the tumor suppressor role of TXNIP in androgen-dependent prostate cancer cells.

### TXNIP recovery after ADT is essential for a successful response in mouse models

**Fig. 5:**
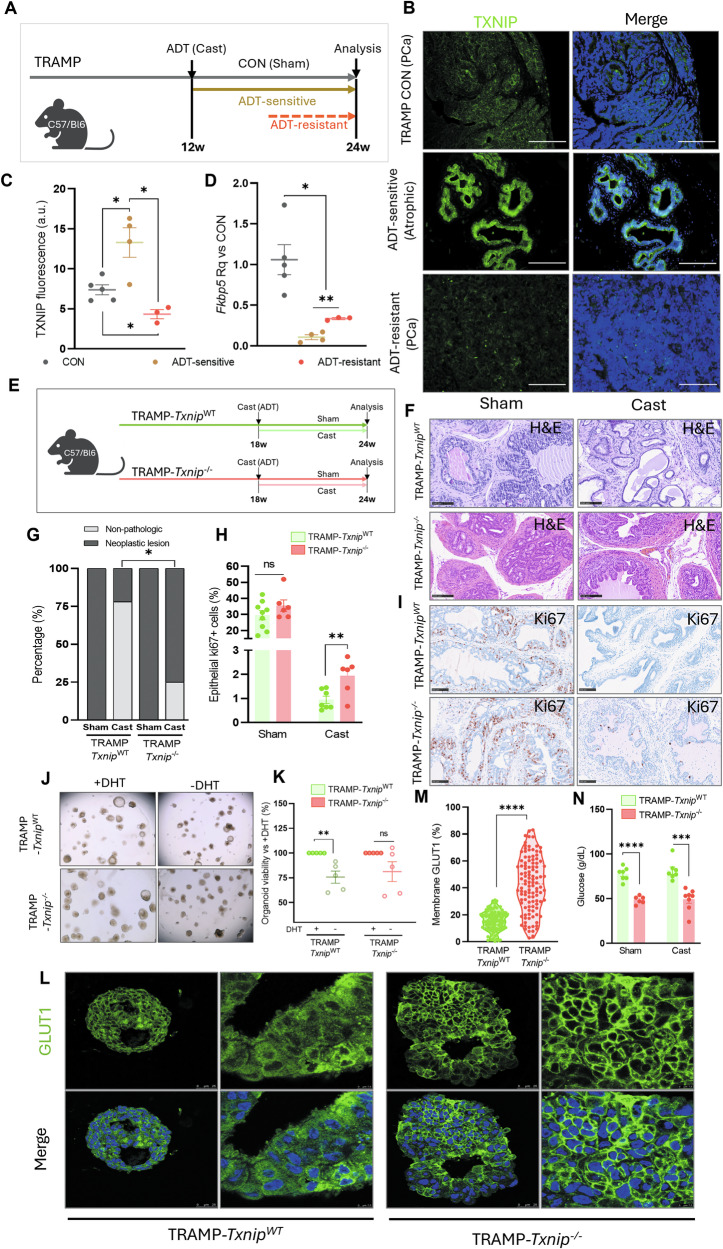
TXNIP recovery after ADT is essential for a successful therapy. **A** Experimental design included untreated mice (Sham) and ADT-treated mice (Cast). Weeks after ADT, spontaneous resistant mice arose (ADT-resistant), while ADT-sensitive finished the experiment at 24 weeks without relapses. **B** Representative micrographs of TXNIP detection (green) in prostate tissue of TRAMP control showing tumoral morphology (TRAMP CON), TRAMP ADT-sensitive after castration showing non-tumoral atrophic glands, and TRAMP ADT-resistant after the same time of castration, showing a poorly differentiated tumoral histology. DAPI counterstaining in blue. 20x magnification, scale bar 100 μm. **C** Quantification of the TXNIP immunofluorescence. **D** mRNA relative expression of *Fkbp5* in prostate tissue from the previous three groups, related to TRAMP CON. **E** Experimental design. Mice from genotypes TRAMP*-Txnip*^WT^ (PCa with competent TXNIP) and TRAMP*-Txnip*^−/−^ (PCa lacking TXNIP) were either treated for ADT by castration (Cast) or Sham-operated as controls (Sham) at 18 weeks-old. 6 weeks later, mice were euthanized and prostates analyzed. **F** Representative hematoxylin eosin staining of prostates from TRAMP-*Txnip*^WT^ and TRAMP*-Txnip*^*−/−*^ castrated (Cast) or control (Sham). Magnification 20x, scale bar 100 μm. **G** Percentage of samples diagnosed with tumoral pathologies (neoplastic lesions) or without tumoral lesions (non-pathologic) from prostates of TRAMP-*Txnip*^WT^ and TRAMP*-Txnip*^*−/−*^ castrated (Cast) or control (Sham). TRAMP-*Txnip*^WT^ Sham *N* = 10, Cast *N* = 9; TRAMP*-Txnip*^*−/−*^ Sham *N* = 6, Cast = 8 (Chi-square test). **H** Quantification of percentage of positive Ki67 cells per total number of cells in the prostate gland epithelia in the 4 groups indicated in (**G**). **I** Representative images of Ki67 immunostaining in prostates from TRAMP-*Txnip*^WT^ and TRAMP*-Txnip*^*−/−*^ castrated (Cast) or control (Sham). Magnification 20x, scale bar 100 μm. **J** Bright-field images of organoids TRAMP*-Txnip*^WT^ and TRAMP*-Txnip*^−/−^ under control (+DHT) or ADT conditions (−DHT). Magnification 20x, scale bar 100 μm. **K**. Determination of the viability of organoid lines in ADT conditions (-DHT) compared to control (+DHT). Mean ± SEM of *N* = 5 independent organoid lines per genotype seeded in replicates. **L** Representative images of GLUT1 immunostaining (green) in organoids TRAMP*-Txnip*^WT^ and TRAMP*-Txnip*^−/−^. Left panel of each group magnification 63x, scale bar 25 μm. Right panels of each group represent a detail of the organoid pictured on the left. Magnification 150x, scale bar 7.5 μm. Low panels show the merge of GLUT1 stain and DAPI counterstain in blue. **M** Representation of the percentage of GLUT1 found in membrane from the total GLUT1 detected in the cells of organoids TRAMP*-Txnip*^WT^ and TRAMP*-Txnip*^−/−^. **N** Circulating glucose levels in TRAMP-*Txnip*^WT^ and TRAMP*-Txnip*^*−/−*^ mice control (Sham) or castrated (Cast). **C**, **D**, **I**, **J**, **L**, **M** Two-tailed unpaired T-test. N of each experiment is indicated in the respective graph. *p-value < 0.05; ** *p*-value < 0.01; ***p-value < 0.001; ****p-value < 0.0001.

We next aimed to confirm the TXNIP impact on ADT treatment. We performed a surgical orchiectomy in 12 weeks-old TRAMP mice at early stages of tumor development. By week 24, two distinct groups emerged among treated animals: therapy-sensitive and therapy-resistant mice (Fig. 5A). Efficacy of castration was confirmed in both groups (Fig. S5A). Responsive tumors displayed atrophic prostatic glands and showed more than a two-fold increase in TXNIP levels (*p* < 0.05 vs. CON; Fig. 5B, C). Strikingly, ADT-resistant tumors, characterized by poorly differentiated morphology, displayed nearly undetectable TXNIP levels, significantly lower than either ADT-sensitive tumors and controls (*p* < 0.05; Fig. 5B, C).

Since AR activity in absence of androgens is one of the most common mechanisms of therapy resistance, we evaluated AR activity in ADT-resistant mice. We performed an RT-qPCR of the AR-reporter gene *Fkbp5* in prostate samples [32]. *Fkbp5* showed a dramatic reduction in expression in ADT-sensitive animals, but ADT-resistant tumors exhibited a threefold increase (*p* < 0.01; Fig. 5D), indicating a partial recovery of AR signaling. An increase in *Txnip* levels was also observed in the transgenic prostate adenocarcinoma mouse model Pten upon ADT. We analyzed single cell RNAseq data available from Terzic et al. [33], where surgical castration increased both the expression levels of *Txnip* and the proportion of cells that express the gene (log2FC = 1.012, Pten^Sham^ fraction of cells positive *Txnip* = 0.498 vs. Pten^ADT^ *Txnip* = 0.591, *p*-value = 0,0187).

Thus, to confirm the mechanistic role of TXNIP in the process of ADT resistance, we generated a model of PCa-*Txnip* knock-out mouse by crossing PCa TRAMP C57/Bl6 mice with a systemic *Txnip* knock-out (*Txnip*^−/−^) C57/Bl6 model [19]. The absence of Txnip (Fig. S5B) was confirmed along with features previously described in mouse embryonic fibroblast (MEF), such as accelerated growth rate and earlier senescence onset (Fig. S5C–E). TRAMP-*Txnip*^−/−^ mice showed similar health status and body weight as their TRAMP-*Txnip*^WT^ counterparts (Fig. S5F, G).

Mice from both strains TRAMP-*Txnip*^WT^ and TRAMP-*Txnip*^−/−^, were either surgically castrated or sham-operated at 18 weeks, and they were sacrificed at 24 weeks (Fig. 5E). There were no differences histopathologically between Sham groups (Fig. 5F, G). Notably, castrated TRAMP-*Txnip*^WT^ mice developed fewer neoplastic lesions compared to castrated TRAMP-*Txnip*^−/−^ mice. (22% vs 75%) (p-value < 0.05; Fig. 5G). Immunohistochemistry for the proliferation marker Ki67 further supported this increase of neoplastic lesions in TRAMP-*Txnip*^−/−^ after castration (Fig. 5H, I, *p* < 0.01). Regarding the immune TME, TRAMP-*Txnip*^−/−^ showed more T cells (CD3^+^) than TRAMP-*Txnip*^WT^ in the prostate in Sham conditions (*p* < 0.05, Fig. S5H, I). Interestingly, a strong and significant increase in T cells was observed in TXNIP-competent TRAMP mice after castration (*p* < 0.001; Fig. SH, I); however, this increase was not observed in TXNIP-deficient TRAMP mice, which may also reflect the lower androgen dependency shown by TRAMP-*Txnip*^−/−^ mice and contribute to resistance to the therapy.

We next established 10 independent organoid lines (five TRAMP-*Txnip*^WT^ and five TRAMP-*Txnip*^−/−^). Organoid morphology was heterogeneous, ranging from (poly)cystic to full (Fig. S5J). Noteworthy, 3 out of 5 TRAMP-*Txnip*^−/−^ mice showed poorly differentiated tumors (Fig. S5J “parental tumor” and S5K). Viability assays revealed that organoids from TRAMP-*Txnip*^WT^ showed a significant viability reduction in absence of androgens whereas TRAMP-*Txnip*^−/−^ organoids showed no changes (*p* < 0.01 and *p* = 0.1 respectively; Fig. 5J, K), indicating that low levels of TXNIP confers less sensitivity to ADT mimicking treatment.

Given TXNIP control of GLUT1 location, we examined GLUT1 distribution in organoids. Membrane-located GLUT1 accounted for 15.5% of the total GLUT1 in TRAMP-*Txnip*^WT^ cells, while TRAMP-*Txnip*^−/−^ organoids showed 42.6% of GLUT1 in the membrane (*p* < 0.0001; Fig. 5L, M). This change in GLUT1 would increase glucose uptake and might contribute to the lower levels of circulating glucose observed in TRAMP-*Txnip*^−/−^ mice in both sham (*p* < 0.0001) and castrated (*p* < 0.001) conditions compared to TRAMP-*Txnip*^WT^ (Fig. 5N).

Previous works have linked epithelial basal cells with glycolytic metabolism [33] and increased resistance to ADT [34, 35], while the transition to the luminal phenotype was associated with an increase in oxidative metabolism [33]. Thus, we sought to identify differences in epithelial populations beyond TXNIP levels that could affect ADT resistance. First, we observed that cells from organoids used by Giafaglione et al. [33] with blocked basal-luminal transition showed higher levels of TXNIP expression, while cells from luminal differentiated organoids lowered TXNIP levels as observed by scRNA-seq data (Fig. 5SL). Furthermore, RNAseq analysis from basal and luminal cells in human and mouse prostates further confirmed that luminal cells have increased TXNIP levels than basal cells [36, 37] (Fig. S5M, *p* = 0.07 and S5N, *p* < 0.01, respectively). In light of these results, we assessed CK8/CK5 markers for luminal/basal identity, the organoid showed a majority of mixed phenotypes in both TRAMP-*Txnip*^WT^ and TRAMP-*Txnip*^−/−^, with no differences in the distribution of epithelial populations between the two groups (Fig. S5O, P), discarding differential enrichment in basal cells in Txnip-deficient organoids.

### The loss of TXNIP during ADT is present in prostate tumor relapses

We finally sought to study TXNIP during ADT in human PCa. Using the cohort of Grasso et al. [28], which allows us to group the data in androgen-sensitive PCa or CRPC, we found that *TXNIP* is downregulated in both androgen dependent PCa (*p* < 0.01) and CRPC (*p* < 0.001) compared to normal tissue. Moreover, CRPC samples had significantly lower *TXNIP* levels than PCa (*p* < 0.05) (Fig. S6A). Besides, *SLC2A1* (GLUT1) expression was increased (*p* < 0.01) and *CDKN1D* (p27^kip1^) expression was decreased (*p* < 0.01) in CRPC samples only (Fig. S6B, C). The AR-reporter gene *FKBP5* was substantially upregulated in CRPC samples (*p* < 0.0001), negatively correlating with *TXNIP* expression, consistent with our earlier findings (Fig. S6D).

**Fig. 6:**
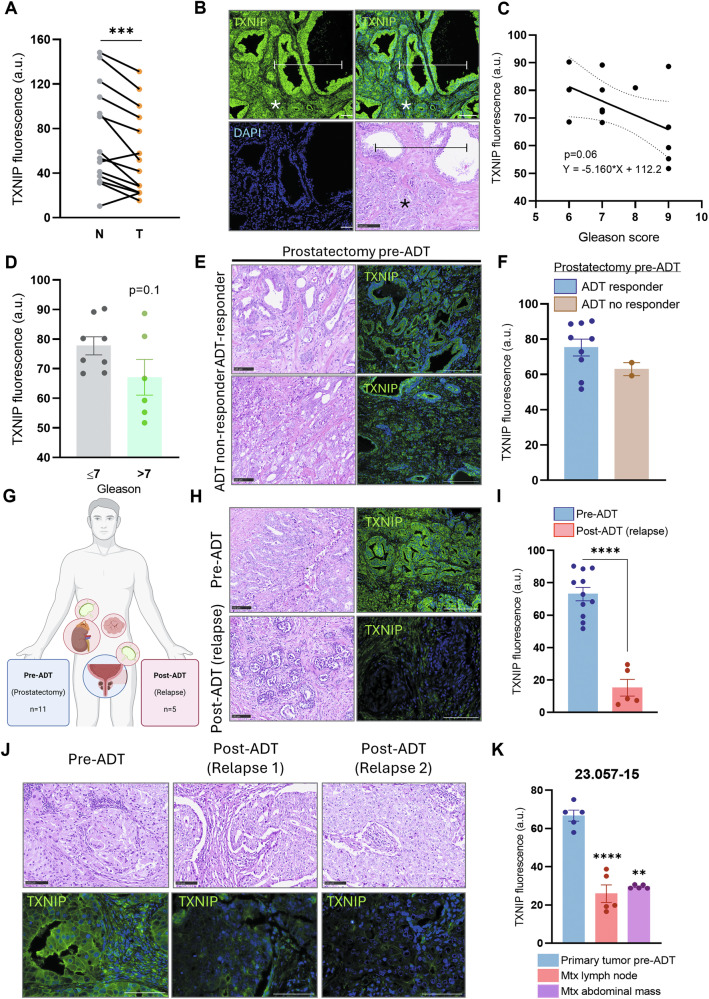
The loss of TXNIP during ADT is a clinically relevant event. **A** Comparison of the TXNIP protein levels studied by immunofluorescence intensity (a.u.) between paired non-tumoral (N) and tumoral (T) tissues in samples from PCa patients. **B** Representative images of non-tumoral (N, marked by bars) and tumoral (T, marked by asterisks) areas used for TXNIP detection showed in “A” and hematoxylin-eosin of an analogous zone. 20x magnification, scale bars 50 μm. **C** Correlation between TXNIP intensity and Gleason score from patient samples. **D** Comparison of TXNIP levels between tumors grouped into more differentiated (Gleason ≤ 7) and more undifferentiated (Gleason >7). **E** Patients simples treated with ADT were grouped into responders (ADT-responders) and not responders (ADT no responder), representative images of Hematoxylin-eosin and TXNIP immunofluorescence in prostatectomies before ADT are shown. 20x magnification, scale bars 100 μm. **F** Cuantification of TXNIP levels in simples from (**E**). **G** Depiction of the 9 samples obtained before ADT and the 5 samples post-ADT. As shown, post-ADT samples are tumoral relapses in two lymph nodes, one that formed an abdominal mass, one in an adrenal gland, and one in the prostate. **H** Representative images of hematoxylin-eosin and TXNIP immunofluorescence in a sample pre-ADT and post-ADT (relapse). 20x magnification, scale bars 100 μm. **I** TXNIP levels as fluorescence intensity in pre-ADT and post-ADT (relapse) samples. **J** Study of a patient with available tumoral tissue before and after ADT. Lower panels show TXNIP detection with very low levels of fluorescence in the ADT relapses. 20x magnification, scale bars 100 μm. **K** Quantification of the TXNIP levels in the samples from patient 23.057-15 with pre-ADT and two post-ADT (relapse) samples. Data shown as mean ± SEM. **A**, **D**, **I**, **K** two-tailed unpaired *T*-test. **F** two-tailed unpaired Mann–Whitney test. *p < 0.05; **p < 0.01; ***p < 0.001; ****p < 0.0001.

Analyzing the histopathological samples from PCa patients, we confirmed the decrease of TXNIP protein in tumor tissue compared to the adjacent benign tissue (*p* < 0.001; Fig. 6A, B). Similarly to the animal model, TXNIP levels and Gleason score showed an inverse trend, on the verge of significance (Fig. 6C, D, *p* = 0.06 and 0.1, respectively). Sixteen patients had undergone ADT, and clinical response follow-up data were also available (Table SII). The AR status was characterized in these samples, with a heterogeneous expression pattern. In pre-ADT samples, the majority displayed more than 50% of AR-positive cells, with some cases reaching 90–100%, while three samples exhibited less than 40% positivity. In post-ADT relapse samples, two cases showed strong AR staining in nearly all tumor cells, one had approximately 40% AR-positive cells, and two were completely negative for AR expression. All AR-positive samples showed higher nuclear/cytoplasmic AR ratio (between 1.5 and 2.5 nuclear fold-change). No significant differences in TXNIP levels in prostate tissue were observed before treatment between responders and non-responders (Figs. 6E, F and S6E). We further analyzed TXNIP levels in samples after ADT, including some obtained from distant metastasis (Fig. 6G). A dramatic reduction in TXNIP was observed in relapse samples after ADT (*p* < 0.0001; Fig. 6H, I). For one of the patients on relapse, samples from the primary tumor and 2 metastatic sites were available. TXNIP levels in the primary tumor pre-ADT were significantly higher than in relapse samples from the lymph node (*p* < 0.0001) and abdominal mass (*p* < 0.01) (Fig. 6J, K). These results indicate that TXNIP loss during ADT might be a clinically relevant event.

In GLUT1 protein levels, no changes were observed between pre- and post-ADT relapse samples or between prostatectomy samples from responders and non-responders (Fig. S6F, G). Similarly, circulating glucose levels showed no significant differences (Table SVII).

## Discussion

This study highlights the crucial role of TXNIP in prostate tissue homeostasis and in ADT response in PCa. Our research demonstrates that TXNIP controls metabolic and cell cycle features and that it might control the transition to androgen independence. To our knowledge, this is the first time that TXNIP is suggested as a major suppressor of androgen-independence transition in PCa.

TXNIP has been proposed as a tumor suppressor gene in several cancers, including breast [38], melanoma [39], and lung [40]. However, its role appears to be tissue-specific, as some cancers, such as kidney [41] or liver [42], exhibit higher TXNIP levels associated with poorer survival.

The key findings about TXNIP in prostate tissue and PCa in our work can be summarized as follows: First, *TXNIP* gene responds to androgens. The expression of *TXNIP* is decreased by DHT, while AR inhibition significantly increased *TXNIP* levels. Using PROMO 3.0 [30], we predicted several androgen receptor binding sites (AREs) in the *TXNIP* promoter, which are conserved across human, mouse, and rat species. This observation is consistent with previous reports describing direct AR binding to the *TXNIP* gene in pancreatic β-cells [29]. We functionally validated the direct interaction between AR and the *TXNIP* promoter by ChIP in prostate cancer cells. To the best of our knowledge, this is the first report of such binding in the context of prostate cancer, providing novel insight into AR-mediated transcriptional regulation in this tissue.

Second, TXNIP effects on cell cycle in PCa. LNCaP cells overexpressing TXNIP exhibited reduced basal growth rates and diminished response to DHT stimulation compared to control cells. Despite TXNIP is a pro-apoptotic protein, no differences in cell death were found. Instead, cell cycle arrest along with an increase in p27^kip1^ in LNCaP TXNIP cells was observed, as well as an increase in senescence. Indeed, p27^kip1^ regulates the cell cycle by halting the G1-to-S transition [43]. In PCa, low levels of p27^kip1^ are predictive of treatment failure [44]. p27^kip1^ is regulated mainly by protein stability [43], and TXNIP stabilizes p27^kip1^ by direct binding in other tissues [45].

Growth was also evaluated together with invasiveness in 3D culture conditions. LNCaP TXNIP cells formed fewer colonies in Matrigel and smaller, less solid spheroids. In contrast, androgen-independent PC-3 cells overexpressing TXNIP showed no changes in growth or apoptosis. Since PC-3 cells are androgen-independent, altogether, our results indicate that TXNIP effects depend on functional AR signaling. Noteworthy, Xie et al. [46] described proapoptotic and cell-cycle arrest effects of TXNIP on PC-3 cell; however, their experiments were performed directly after transfection, which can alone produce a pro-apoptotic effect that might be increased by TXNIP, while ours were performed in a PC-3 population with long term stable transfection of TXNIP. Therefore, further clarification is needed about TXNIP overexpression in androgen-independent cells at long and short term basis.

Third, metabolic rearrangement. PCa progression is marked by unique metabolic adaptations, including an “inverse Warburg effect” [47]. We studied both glucose and redox metabolism. No changes were observed in the redox state, either cytoplasmic or mitochondrial, at the enzymatic level or in the concentration of free radicals, except for an increase in GPX4 protein. This enzyme is critical for scavenging lipid peroxides, preventing ferroptosis in tumor cells, and TXNIP has recently been regarded as a pro-ferroptotic protein [48, 49]. However, changes in protein levels alone should be interpreted with caution, and further investigation of its enzymatic activity is required to draw more definitive conclusions on GPX4. No changes in cell viability were observed between LNCaP Empty or LNCaP overexpressing TXNIP.

Conversely, a dramatic drop in glycolysis and glucose metabolism was observed, probably due to GLUT1 sequestration in the cytoplasm, impairing glucose uptake. It was already known that GLUT1 was internalized by TXNIP in clathrin-coated vesicles [9] and that its subcellular location is important in tumor progression [50]; however, its importance is not so well studied in the context of PCa-ADT. In TRAMP-*Txnip*^−/−^ organoids, the vast majority of GLUT1 signal was in the membrane. By contrast, LNCaP TXNIP-overexpressing cells had most of the GLUT1 signal in the cytoplasm. TXNIP-overexpressing cells show a shift to glutamine metabolism and a higher dependency for this amino acid, probably as a substitute for the lack of glucose. A decrease in glycolysis-derived species was observed, along with an increase in species derived from other carbon sources, such as glutamate, and more notably αKG, the intermediate between glutamine-glutamate and the TCA cycle that showed a significant proportion of unlabeled carbon in LNCaP TXNIP cells and a strong increase in the total concentration of the metabolite, which could be fueling LNCaP TXNIP metabolism [51].

We conclude that the recovery of TXNIP through ADT derives the glycolytic metabolism of PCa cells towards a more oxidative phenotype, which actually resembles the first, androgen-sensitive stages of the disease [52], and downregulates the proliferation and survival pathways. In fact, we showed that LNCaP cells incubated with the anti-androgen CDX showed the same profile as TXNIP-overexpressing cells, with a decrease in glycolysis. As an example, the oncogene c-Myc is a known inhibitor of TXNIP in multiple cancers, TXNIP_low_/Myc_high_ induces metabolic reprogramming and is a marker of poor prognosis in breast cancer [53], and therapy resistance [54, 55].

Finally, the potential role of TXNIP in ADT was also demonstrated. It is remarkable the increase of TXNIP in mice that successfully responded to ADT compared to non-responders and primary tumor. This suggests that the failure to recover TXNIP is a crucial step in the development of ADT resistance. This theory is strongly supported by the observation that TRAMP-*Txnip*^−/−^ mice showed a worse response to ADT treatment, with more neoplastic lesions and increased cell proliferation in the prostate. Furthermore, organoids from TRAMP-*Txnip*^−/−^ prostates did not show decreased viability upon ADT.

Our results in a cohort of patients indicate that TXNIP protein levels are higher in healthy tissue compared with tumor tissue from the same individual. We also observed a decrease of TXNIP protein along tumor progression, correlating with Gleason score, similarly to what was observed in the TRAMP model. We confirmed the decrease of *TXNIP* in PCa described in large RNA sequencing studies from four open databases of prostate cancer patients.

Notably, all tumor relapse samples after ADT therapy exhibited very low TXNIP levels, indicating that low levels of TXNIP are needed for tumor recurrence; however, TXNIP levels pre-therapy did not forecast therapy response, although a trend was observed. Both metrics must be validated in larger patient cohorts.

The cell cycle arrest mediated by p27^kip1^ and the decrease of GLUT1 were confirmed in the patient data set from Grasso et al. [28] which allows grouping by androgen-sensitive PCa and mCRPC. The group mCRPC has lower *TXNIP* and *CDKN1D* (p27^kip1^) expression than localized PCa, along with higher levels of *SLC2A1* (GLUT1) and the AR-response gene *FKBP5*. In the cohort of histological human samples, no differences were observed in GLUT1, although our data is obtained from a small cohort due to the difficulty of accessing post-ADT human tissue for histological analysis. Larger cohorts might be more informative for GLUT1 studies.

Our work is particularly relevant in the context of clinical diabetes. Diabetes, which is a risk factor for several tumor types, prevents PCa [56]. Significant efforts are being made to target TXNIP in diabetic pathologies. The TXNIP inhibitors TIX100 and Verapamil are approved for or under clinical trials [57] (ClinicalTrials.gov ID NCT06800729 and NCT02372253) to test its ability to prevent T1D. While these studies are promising, our findings strongly suggest that TXNIP inhibition to treat T1D should be avoided in PCa patients undergoing ADT.

In summary, TXNIP emerges in our work as a significant factor for tumor response to ADT.

## Supplementary information

Supplementary figures

Supplementary material

Related Manuscript File

## Data Availability

The data analyzed in this study were obtained from EMBL-EBI Array Express at E-TABM-26 and Gene Expression Omnibus (GEO) at GSE80609, GSE35988, GSE46602, GSE216158, GSE67070, and GSE122367. Data from scRNA-seq can be found at Single Cell Portal #SCP1234. *TXNIP* expression was given either as log2 expression vs non-tumoral samples or as normalized FPKMs, and the expression data were analyzed with Prism 8 software.
