# Supplementary material for: TXNIP upregulation controls metabolism and cell cycle during androgen deprivation therapy in prostate cancer
Source: Cell Death Dis. 2025 Nov 10;16(1):817. doi: 10.1038/s41419-025-08128-4 (PMC12603336; doi:10.1038/s41419-025-08128-4)
Supplement: Supplementary file 1 — Supplementary figures [file 41419_2025_8128_MOESM1_ESM.pdf]

TXNIP upregulation controls metabolism and cell cycle during androgen deprivation therapy in prostate cancer.

Sergio Alcon-Rodriguez[1,2,3], Juan C. Mayo[1,2,3], Pedro Gonzalez-Menendez[1,2,3], Iván Fernandez-Vega[3,4,5], David Hevia[1,2,3], Sheila Fernandez-Vega[1,2,3], Alba Moran-Alvarez[1,2,3], Daniela Pineda-Cevallos[3,6], Miguel Alvarez-Múgica[7], Pablo Rodríguez-González[3,6], Belen Garcia-Soler[1,2,3], Jorge Zamora[8,9,10], Jose M. C. Tubio[8,9,10], Rosa M. Sainz[1,2,3]* & Isabel Quiros-Gonzalez[1,2,3]*.

[1] Department of Morphology and Cell Biology, School of Medicine, University of Oviedo, Spain

[2] Oncology Institute of Principado de Asturias (IUOPA), Oviedo, Spain

[3] Health Research Institute of Principado de Asturias (ISPA), Oviedo, Spain

[4] Department of Pathology, Hospital Universitario Central de Asturias (HUCA), Oviedo, Spain

[5] Biobank of the Principality of Asturias (BioPA), Oviedo, Spain.

[6] Department of Physical and Analytical Chemistry, School of Chemistry, University of Oviedo, Spain

[7] Department of Urology, Hospital Valle del Nalón, Langreo, Spain

[8] Mobile Genomes, Centre for Research in Molecular Medicine and Chronic Diseases (CIMUS), Universidad de Santiago de Compostela, Santiago de Compostela, Spain.

[9] Instituto de Investigaciones Sanitarias de Santiago de Compostela (IDIS), Santiago de Compostela, Spain.

[10] Department of Zoology, Genetics and Physical Anthropology, Universidad de Santiago de Compostela, Santiago de Compostela, Spain.

SUPPLEMENTARY FIGURES

Suppl. Fig 1.

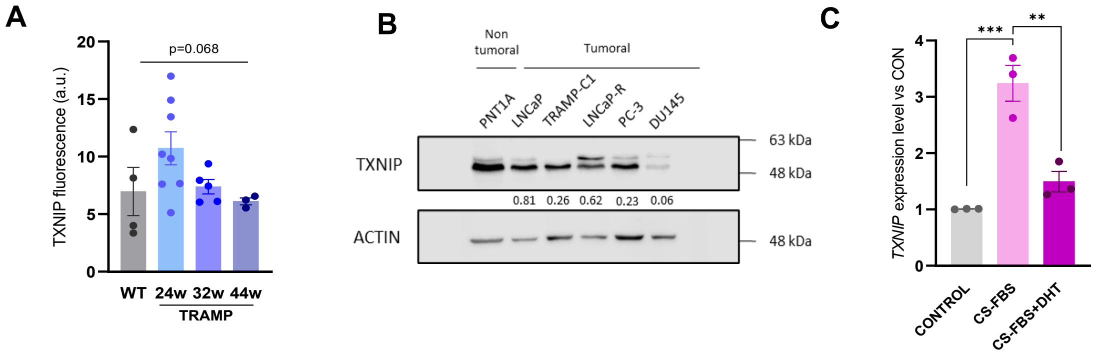

Supplemmentary Figure S1. A. TXNIP abundance assessed by fluorescence quantification in the prostate of WT and TRAMP (24, 32 and 44 weeks). One-way ANOVA followed by Tukey's multiple comparisons test. B. Immunoblotting of TXNIP from protein extracts of the indicated prostate cell lines. Relative optical density values normalized vs ACTIN as load control are shown in the respective columns C. TXNIP RNA relative expression of LNCaP cells cultured under control (complete FBS), charcoal-stripped FBS (CS-FBS) to mimic ADT conditions, or CS-FBS+DHT to restore androgen signaling (two-tailed unpaired T-test vs CON). Data shown as mean±SEM of three different experiments. ** p<0.01, *** p<0.001.

Suppl. Fig 2.

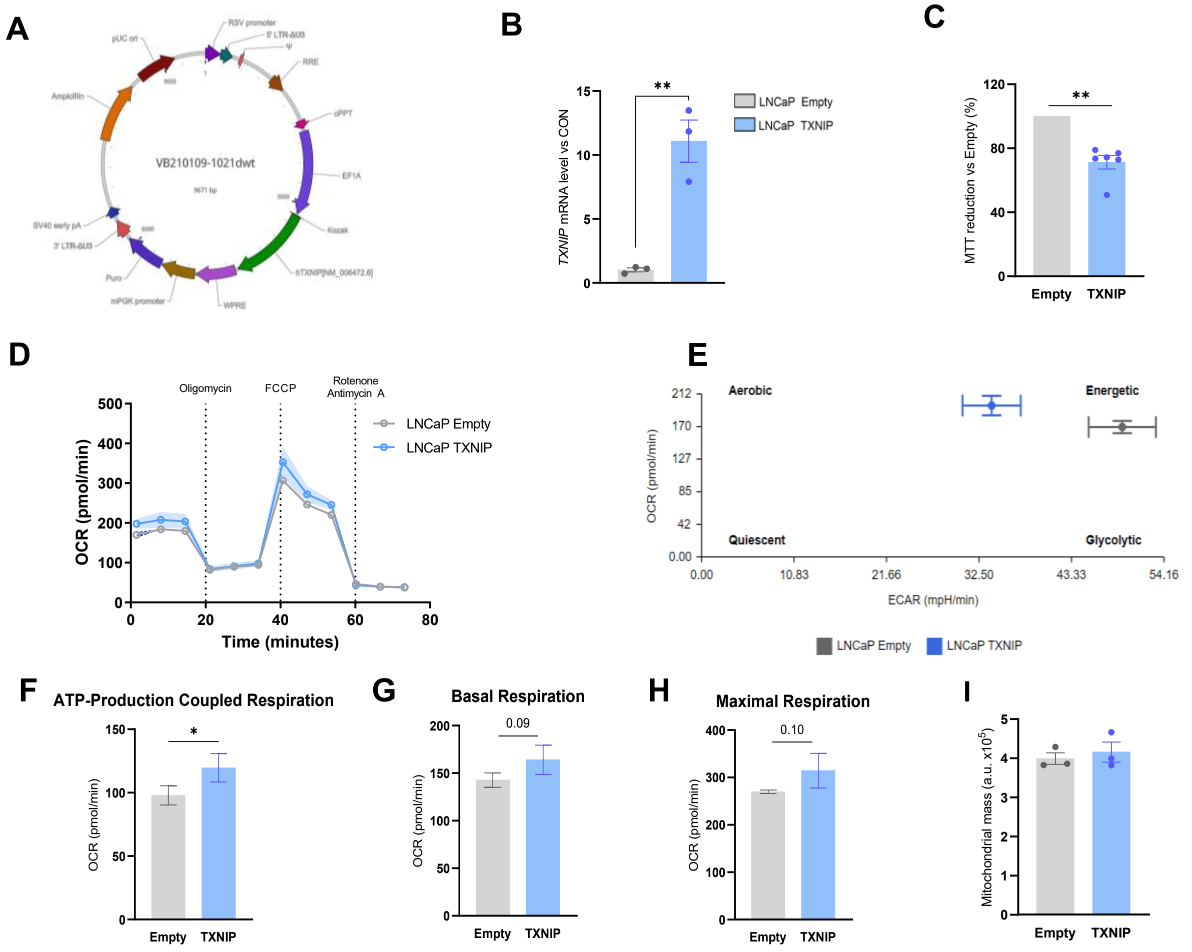

Supplementary Figure S2. A. Lentiviral vector containing TXNIP cDNA used for transduction of LNCaP and PC-3 cell lines. B. TXNIP overexpression in transduced LNCaP cell lines was confirmed by RT-qPCR (two-tailed unpaired t-test). C. MTT reduction capacity of LNCaP Empty and LNCaP TXNIP cells. Data as mean ±SEM of percentage to Empty from N=6 independent experiments. D. Oxygen Consumption Ratio (OCR) was measured in LNCaP TXNIP cells following injections of oligomycin, FCCP and rotenone/antimycin A as indicated. E. Energy diagram representing ECAR vs OCR of LNCaP Empty and LNCaP TXNIP cells. F. ATP produced derived from respiration in LNCaP Empty and LNCaP TXNIP cells. G. Basal respiration of LNCaP Empty and TXNIP. H. Maximal respiration of LNCaP Empty and LNCaP TXNIP cells. I. MitoGreen fluorescence quantification for mitochondrial biomass determination expressed as arbitrary units in LNCaP Empty and LNCaP TXNIP. F-H two-tailed unpaired T-test, mean±SEM, N=3 technical replicates. I mean±SEM of N=3 technical replicates from a representative experiment, selected from 3 independent experiments. ** $p<0.01$; * $p<0.05$.

Suppl. Fig 3.

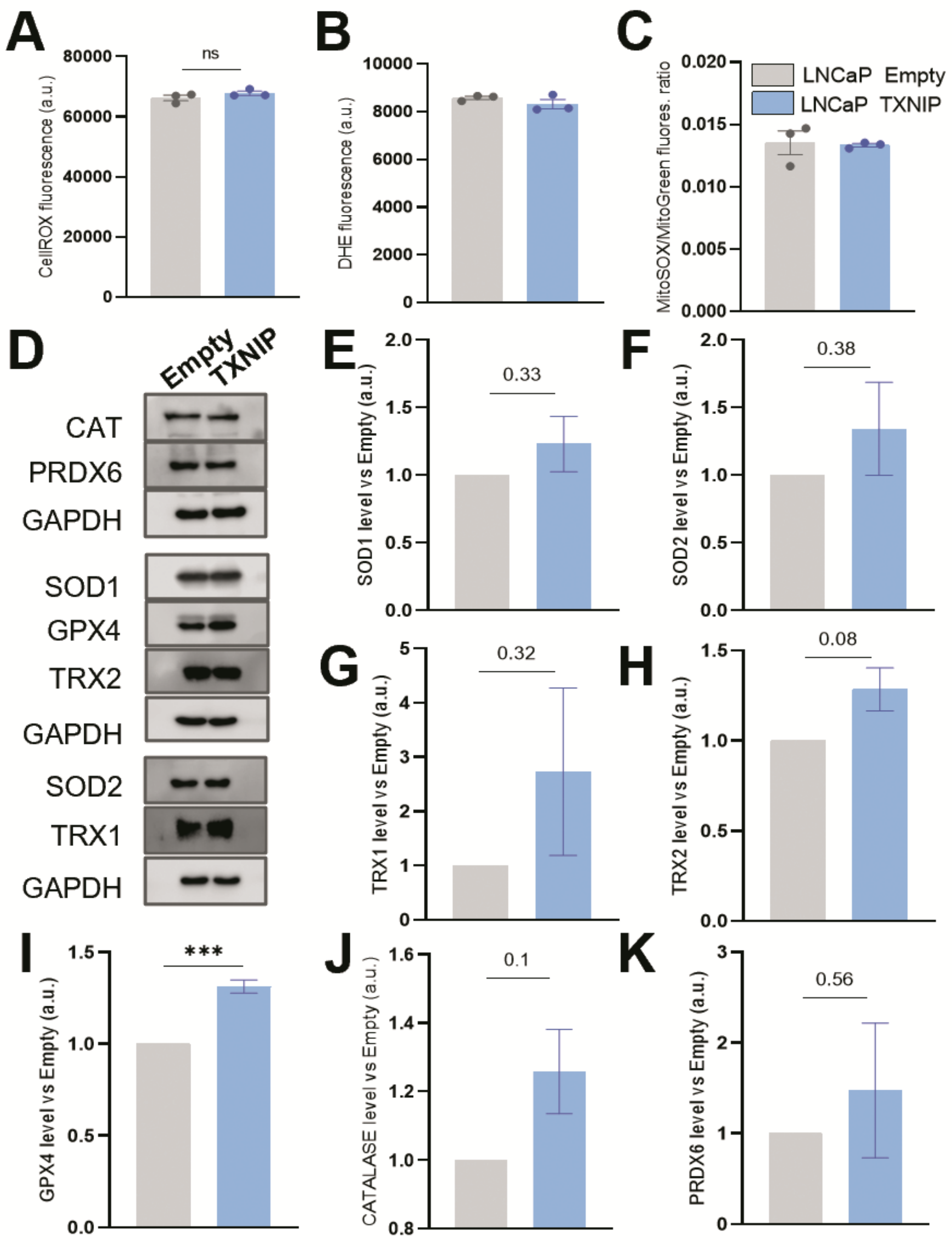

Supplementary Figure S3. A. CellROX fluorescence of LNCaP Empty and LNCaP TXNIP cell lines used for general ROS measurement. B. DHE fluorescence of LNCaP Empty and LNCaP TXNIP cell lines used to assess cytoplasmic O2•-. C. Ratio of MitoSOX/MitoGreen of LNCaP Empty and LNCaP TXNIP cells for assessing mitochondrial O2•-. D. Representative immunoblottings of the indicated antioxidant proteins in LNCaP Empty and LNCaP TXNIP cell lines. GAPDH was used as load control. E-K. Quantification of the protein levels was normalized comparing to LNCaP Empty (E, SOD1; F, SOD2; G, TRX1; H, TRX2; I, GPX4; J, Catalase; K, PRDX6). Data shown as mean±SEM of three independent experiments, unpaired T-test was performed for all comparisons. *** $p<0.001$.

Suppl. Fig 4.

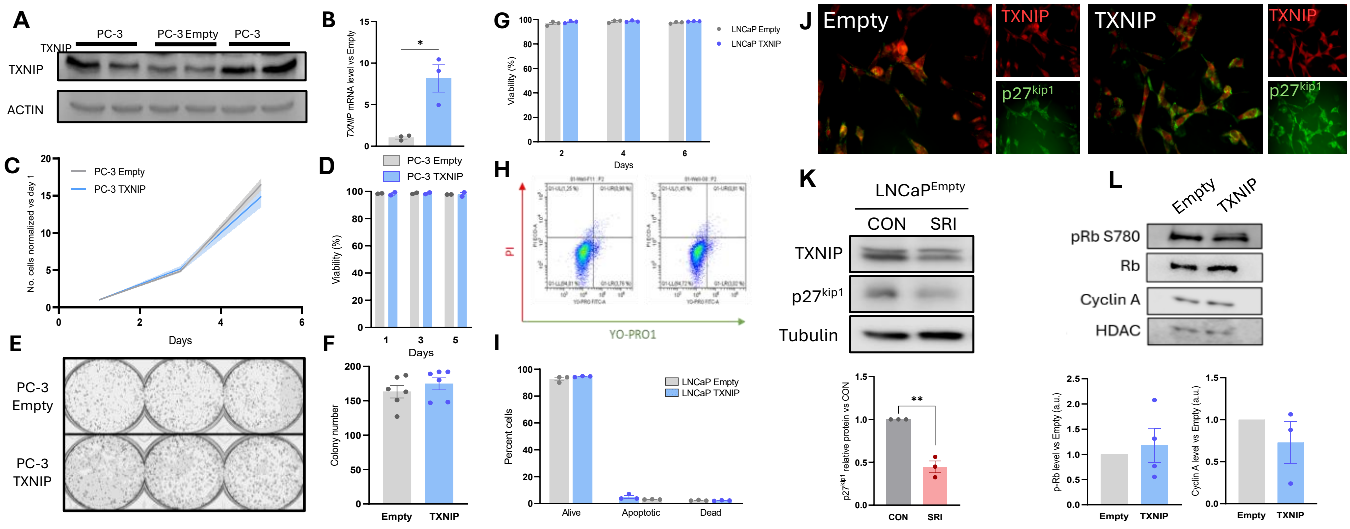

Supplementary Figure S4. A. TXNIP overexpression levels in PC-3 cells were studied in protein. B. TXNIP overexpression in PC-3 cells studied at mRNA level. C. Cell growth rate in PC-3 Empty and PC-3 TXNIP. D. Viability of PC-3 Empty and PC-3 TXNIP assessed by trypan blue exclusion asay. E. Representative images of a colony formation assay in 2D conditions between PC-3 Empty and PC-3 TXNIP cell lines. F. Quantification of colonies formed by PC3 Empty and PC3 TXNIP. G. Viability measured by trypan blue exclusion assay between LNCaP Empty and LNCaP TXNIP across 6 days of culture. H. Cytometry dot plots of the apoptosis experiment assessed by PI/YO-PRO1 cytometry detection in LNCaP Empty and LNCaP TXNIP cells. I. Quantification of the relative number of viable, apoptotic and dead LNCaP Empty and LNCaP TXNIP cells presented as percentage. J. Representative IF of TXNIP (red) and p27kip1 (green) in LNCaP Empty and LNCaP TXNIP. K. Representative immunoblotting showing the TXNIP and p27kip1 reduction upon 30 µM SRI-37330 48h-incubation in LNCaP Empty cells. Below, quantification of the reduction of p27kip1 upon incubation of LNCaP Empty cells with SRI-37330. L. Representative immunoblottings of cell cycle regulators phospho-Rb (Ser780) and Cyclin A in LNCaP Empty and LNCaP TXNIP cells. Below, quantification of p-Rb and Cyclin A levels of both cell lines compared to LNCaP Empty. Data shown as mean±SEM of three independent experiments.

Suppl. Fig 5.

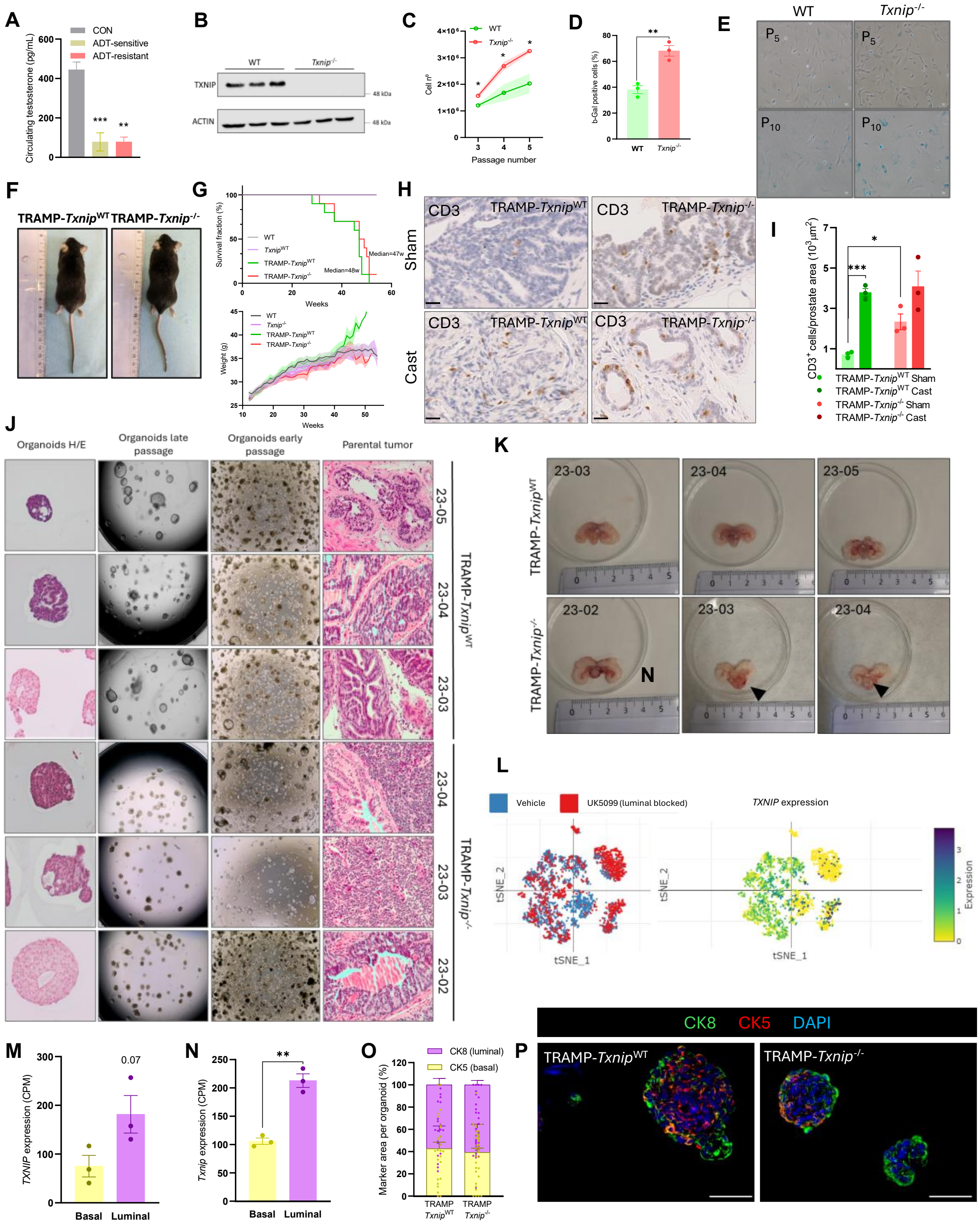

Figure legend: see next page.

Supplementary Figure 5. A. Abscence of circulating androgens in ADT-resistant mice was studied by assessing testosterone levels (pg/mL) in plasma of TRAMP CON, ADT-sensitive and ADT-resistant groups. **B**. Verification of the absence of TXNIP protein in *Txnip*$^{-/-}$ mice by immunoblotting. **C**. Cell growth rate of mouse embryonic fibroblasts (MEFs) derived from *Txnip*$^{-/-}$ and *Txnip*^{WT} , N=3 independent lines of MEFs per genotype. **D**. Quantification of percentage of senescence MEFs at P_{10}. N=3 independent lines of MEFs per genotype. **E**. Representative images of senescence at passage 5 (P_5) and passage 10 (P_{10}) of *Txnip*^{WT} and *Txnip*$^{-/-}$ MEFs. **F**. External aspect of TRAMP-*Txnip*^{WT} and TRAMP-*Txnip*$^{-/-}$ mice. **G**. Overall survival experiments. Top: survival fraction of *Txnip*^{WT} (WT) (N=11), *Txnip*$^{-/-}$ (N=9), TRAMP-*Txnip*^{WT} (N=10) and TRAMP-*Txnip*$^{-/-}$ (N=10) mice (log-rank test TRAMP-*Txnip*^{WT} vs TRAMP-*Txnip*$^{-/-}$). Bottom: body weight of the mentioned groups across the duration of the study. **H** Representative images of CD3 IHC in prostates of TRAMP-*Txnip*^{WT} and TRAMP-*Txnip*$^{-/-}$ sham (CON) and castrated (ADT). 40x magnification, scale bar 20 µm. **I**. Quantification of CD3 positive cells normalized to prostate area in TRAMP-*Txnip*^{WT} and TRAMP-*Txnip*$^{-/-}$ sham (CON) and castrated (ADT) groups. **J**. Example of three out of five organoid lines from TRAMP-*Txnip*^{WT} and TRAMP-*Txnip*$^{-/-}$ genotypes. First column shows H/E for morphology of organoids. 2 and 3 columns show early and late passages of organoids with different confluency. 4 column show the histology of the tumor of origin from which organoid lines were stablished. **K**. Macroscopic appearance of the genitourinary tract of 3 TRAMP-*Txnip*^{WT} and 3 TRAMP-*Txnip*$^{-/-}$ mice from which organoid lines were established. Arrowheads in TRAMP-*Txnip*$^{-/-}$ show solid-appearing structures that correlate with poorly differentiated tumours. **L**, Left, t-SNE plot showing single cells from organoids incubated with either vehicle (cells depicted in blue) or UK5099, an inhibitor of the basal-luminal differentiation (cells depicted in red). Right, t-SNE plot corresponding to the previous single cells on the left graph, colored by *TXNIP* expression (yellow tones less expression, green to blue higher expression). Data from Giafaglione et al, 2023. **M**, *TXNIP* mRNA expression in 3 pairs of benign prostatic basal and luminal populations from prostate tissues of three prostate cancer patients. Data from Zhang D et al, 2016. **N**, *Txnip* mRNA expression in basal and luminal cells isolated from 24-month-old mouse prostates. N=3 biological replicates, each replicate pooled from two mice. Data from Crowell PD et al, 2020. **O**, Distribution of positive area per organoid (as percentage) of CK8 (luminal) and CK5 (basal) markers in five organoid lines from TRAMP-*Txnip*^{WT} (N=25 organoids) and TRAMP-*Txnip*$^{-/-}$ (N=35 organoids) genotypes. **P**, Representative images of TRAMP-*Txnip*^{WT} and TRAMP-*Txnip*$^{-/-}$ organoids stained for CK8 (green), CK5 (red) and DAPI as counterstaining (blue). 40x magnification, scale bar 50 µm. A, C, D, I, M, N, O data as mean±SEM.

Suppl. Fig 6.

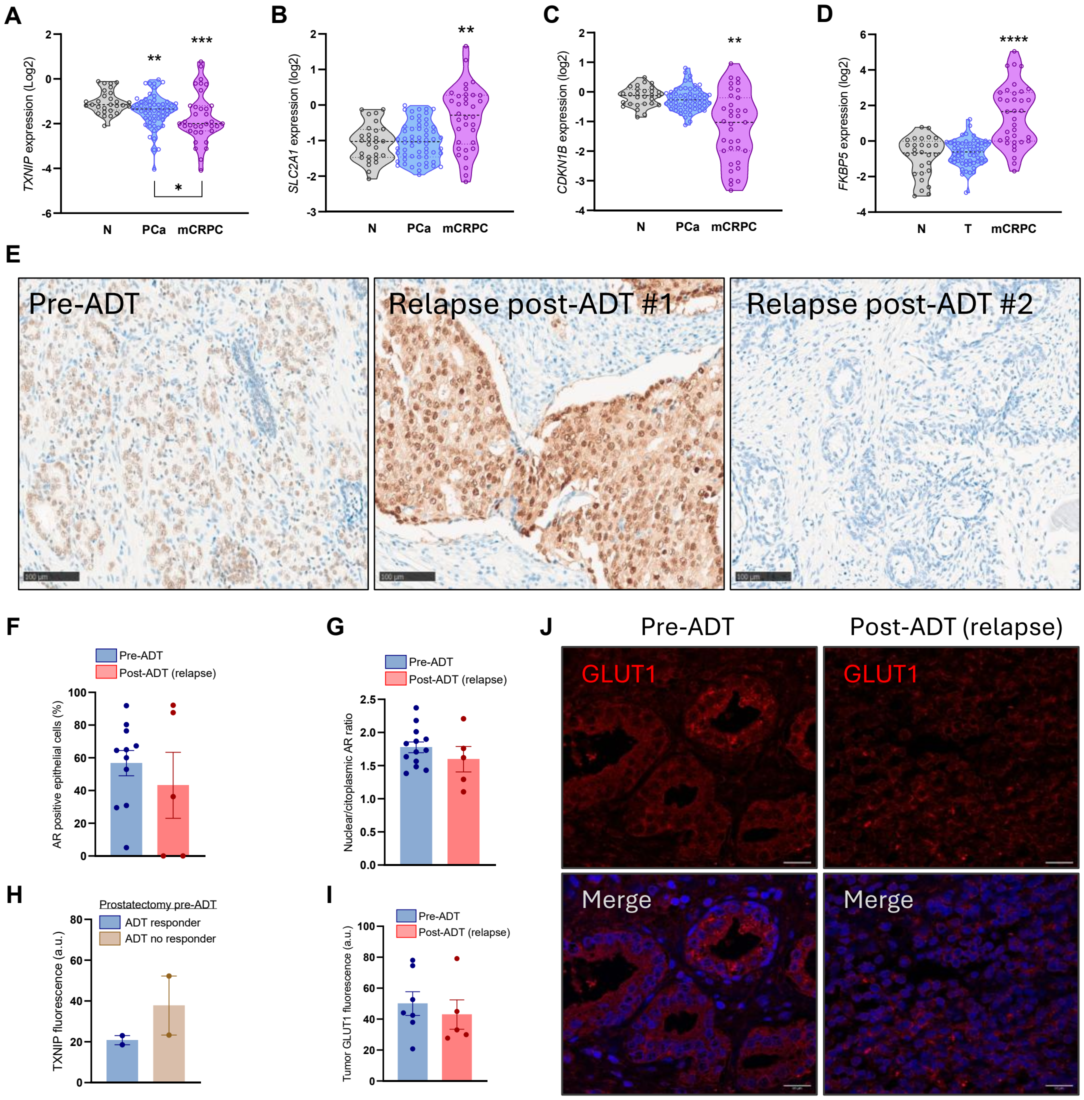

Supplementary Figure 7. A-D. Grasso *et al* cohort separating PCa samples between localized treatment-naïve (PCa) and metastasic CRPC (mCRPC). **A**. *TXNIP* expression as log2 vs normal tissue. **B**. *SLC2A1* (GLUT1) expression as log2 vs normal tissue. **C**. *CDKNB1* (p27^{kip1}) expression as log2 vs normal tissue. **D**. *FKBP5* expression as log2 vs normal tissue. **E**. Representative images of AR IHC in patient samples pre-ADT and two post-ADT showing intense staining (#1) or completely negative staining (#2). 20x magnification, scale bar 100 µm. **F**. Quantification of percent AR+ cells in pre-ADT and post-ADT (relapse) patient samples. **G**. Quantification of nuclear or cytoplasmic location of the AR calculated as the ratio nuclear/cytoplasmic positive area in pre-ADT and post-ADT (relapse) patient samples. **H**. TXNIP fluorescence quantification in prostatectomies pre-ADT from patients who correctly responded or not to ADT (Valle del Nalon cohort). **I**. GLUT1 total fluorescence in pre-ADT prostatectomies and in post-ADT relapses of patients. **J**. Representative images of GLUT1 inmmunofluorescence in pre-ADT and post-ADT relapse samples. 63x magnification, scale bar 20 µm.
