# Supplementary material for: TXNIP upregulation controls metabolism and cell cycle during androgen deprivation therapy in prostate cancer
Source: Cell Death Dis. 2025 Nov 10;16(1):817. doi: 10.1038/s41419-025-08128-4 (PMC12603336; doi:10.1038/s41419-025-08128-4)

**TXNIP upregulation controls metabolism and cell cycle during androgen deprivation therapy in prostate cancer.**

Sergio Alcon-Rodriguez1,2,3, Juan C. Mayo1,2,3, Pedro Gonzalez-Menendez1,2,3, Iván Fernandez-Vega3,4,5, David Hevia1,2,3, Sheila Fernandez-Vega1,2,3, Alba Moran-Alvarez1,2,3, Daniela Pineda-Cevallos3,6, Miguel Alvarez-Múgica7, Pablo Rodríguez-González3,6, Belen Garcia-Soler1,2,3, Jorge Zamora8,9,10, Jose M. C. Tubio8,9,10, Rosa M. Sainz1,2,3,* & Isabel Quiros-Gonzalez1,2,3,*.

^1^ Department of Morphology and Cell Biology, School of Medicine, University of Oviedo, Spain

^2^ Oncology Institute of Principado de Asturias (IUOPA), Oviedo, Spain

^3^ Health Research Institute of Principado de Asturias (ISPA), Oviedo, Spain

^4^ Department of Pathology, Hospital Universitario Central de Asturias (HUCA), Oviedo, Spain

^5^ Biobank of the Principality of Asturias (BioPA), Oviedo, Spain.

^6^ Department of Physical and Analytical Chemistry, School of Chemistry, University of Oviedo, Spain

^7^ Department of Urology, Hospital Valle del Nalón, Langreo, Spain

^8^ Mobile Genomes, Centre for Research in Molecular Medicine and Chronic Diseases (CIMUS), Universidad de Santiago de Compostela, Santiago de Compostela, Spain.

^9^ Instituto de Investigaciones Sanitarias de Santiago de Compostela (IDIS), Santiago de Compostela, Spain.

^10^ Department of Zoology, Genetics and Physical Anthropology, Universidad de Santiago de Compostela, Santiago de Compostela, Spain.

SUPPLEMENTARY INFORMATION

Extended information regarding methods.

Supplementary Table I. List of reagents and resources.

Supplementary Table II. Clinical data from patients enrolled in the study.

Supplementary Table III. Antibody clone ID and concentration

Supplementary Table IV. IF conditions.

Supplementary Table V. List of primers and their sequences used in the present work.

Supplementary Table VI. Organoid medium composition (Drost et al, 2016).

Supplementary Table VII. Patient clinical data from prostate cancer cohorts in Fig. 1.

Supplementary Table VIII. Patients glycemia after ADT.

**Material and Methods**

**Animal models and procedures**

Transgenic adenocarcinoma of the mouse prostate [C57BL/6-Tg (TRAMP)8247Ng] [18] and TXNIP^Flox^ (B6;129-Txnip^tm1Rlee^/J) [19] mice were purchased from Jackson Laboratories. TXNIP^Flox^ contains loxP sites flanking exon 1 of the *Txnip* gene, and they were crossed with mice expressing the recombinase Cre to obtain a *Txnip*^-/-^ offspring. Homozygous *Txnip*^-/-^ were then crossed with TRAMP mice. The resulting TRAMP-*Txnip*^+/-^ mice were crossed with *Txnip*^-/-^ to obtain the experimental TRAMP-*Txnip*^-/-^. Mice were bred and maintained in the Animal Facility of University of Oviedo. All animals were exposed to a 12:12 light–dark cycle with food and water *ad libitum* at 22°C and 50% humidity. Genotyping was done 3 weeks after birth with the primers indicated in Supplementary Table V. All the experiments were designed with the approval of the Ethics Committee in Animal Experimentation of the University of Oviedo (PROAE 01/2020 and PROAE 20/2021) and following the European Directive 2012/63/EU. Littermates were randomized right after genotyping into control (Sham) or castration (ADT). A sample size close to 10 mice per group was attempted following standard practice and ensuring reliable results while minimizing animal use (N is indicated as individual points in each graph). ADT model in mice was achieved by bilateral orchiectomy of males at 18 weeks. In short, a small incision of 1 cm was performed in the scrotum of mice under 2% isoflurane anesthesia. Testes were carefully pulled out and removed by cauterization. The incision was closed with Cicastick Veterinary Tissue Adhesive. For sham-operated mice, the same procedure was done without removing the testes. Mice were allowed to recover overnight with the cocktail of analgesics and anti-inflammatories 2 mg/Kg Metacam and 0.1 mg/Kg Bupaq.

During the experiment, body weight and general aspect were registered weekly, and animals were euthanized at 24 weeks old in fasting conditions. Glycemia was assessed using blood from tail bleed extraction and using a conventional glucometer. The prostate tissue was cut in half for molecular biology (snap-frozen) and histological analysis (PFA 4%) following standard procedures for paraffin embedding. Histopathological diagnosis was carried out by a blinded external pathologist.

**Protein extraction and immunoblotting**

Cells were seeded at 25000 cell/mL and left to attach for 48 hours. After the given experimental times, cells were washed with ice-cold PBS and lysed with RIPA buffer (50 mM Tris-HCl, 150 mM NaCl, 0.1% SDS, 1% Igepal, 0.5% sodium deoxycholate) pH 7.4 with CompleteTM Protease inhibitor cocktail, phosphatase inhibitors 1 μM NaF and 200 nM sodium orthovanadate, and 1mM DTT reducing agent. Lysis was allowed for 30 min on ice and then tubes were centrifuged at 15,000 x g 10 min to collect proteins from the supernatant to a new clean tube. A modification from Go and Miller, 1992 was used for nuclear proteins. Briefly, cells collected with trypsin-EDTA and washed with ice-cold PBS were allowed to swell with buffer A (10 mM Hepes pH 7.8, 15 mM KCl, 2 mM MgCl2, 1 mM DTT, 0.1 mM EDTA and the previously indicated protease inhibitors) for 5 min on ice. Cells were centrifuged at 1,400 rpm for 5 min. Cells were then lysed in buffer A with 0.2% NP-40 detergent 10 min. Nuclei were collected by centrifugation at 14,000 rpm 5 min at 4°C and resuspended in Buffer B (20 mM Hepes-KOH pH 7.8, 420 mM NaCl, 1.5 mM MgCl2, 0.2 mM EDTA, 1 mM DTT, 25% glycerol and protease inhibitors) for 15 min on ice. Finally, Buffer C (50 mM KCl, 20 mM Hepes, 0.2 mM EDTA, 20% glycerol and protease inhibitors) was added to the lysed nuclei. Nuclear proteins were collected by centrifugation at 14,000 rpm 30 min at 4°C. Protein concentration was determined by Bradford colorimetric assay.

Protein extracts were loaded and run into acrylamide/polyacrylamide gels. Resolved proteins were transferred to a PVDF membrane with a Trans-Blot Turbo Transfer System (Bio-Rad) following manufacturer’s instructions. Membranes were then blocked with 5% non-fat dry milk for chemiluminescence detection or with Li-Cor blocking buffer for fluorescence detection. Antibodies and dilutions used are specified in Supplementary Table IV. For chemiluminescence detection, secondary antibodies conjugated with HRP were used, and ECL was used as a substrate. Chemiluminescence or fluorescence acquisition was performed in an Odyssey XF (Li-Cor).

Immunostaining

LNCaP cells were seeded at 25000 cell/mL on Thermanox coverslips. After 2 days, cells were washed with PBS and fixed with 4% PFA for 15 min at RT. After washing with PBS, cells were blocked and permeabilized (0.5% BSA, 0.1% Tween TBS) for 30 min. Primary antibodies were incubated overnight at 4°C followed by secondary antibody incubation with Alexa-Fluor 488 1h at RT. Dilutions are specified in Supplementary table III. DAPI (0.5 μg/mL) was used for nuclear counterstaining. Images were taken at 40x and 150x magnification.

For tissue immunofluorescence (IF), 5 µm sections were deparaffined and rehydrated according to standard procedures. Antigen retrieval, blocking and permeabilization steps for the different IF are detailed in Supplementary Table IV. Primary antibodies were incubated overnight at 4°C followed by secondary antibody incubation for 1 hour at RT. Antibody dilutions are provided in Supplementary Table III. DAPI (0.5 µg/mL) was used for nuclear counterstaining. Images were acquired at 20× and 63× magnification. KI67 IHC was performed automatically using the Roche Discovery ULTRA system.

RNA extraction and RT-qPCR

Cells were seeded at 25000 cell/mL, left to attach for 48 hours. After the given experimental times and treatments, cells were scrapped in NZYol and transferred to RNAse-free tubes. For prostate tissue samples, pieces of approximately 50 mg were homogenized with an UltraTurrax in 1 mL of NZYol in RNAse-free tubes.

RNA was purified following the manufacturer’s instructions. One microgram of RNA was used for first-strand cDNA synthesis via RT-PCR. Quantitative PCR (qPCR) was performed using SYBR Green-based probes in a Quant Studio 5 thermocycler (Thermo Fisher). Relative expression levels were calculated using the ^ΔΔ^Ct method. Primers used are specified in Supplementary Table V. For cell culture experiments, three replicates were seeded for each condition, with experiments performed in triplicate. For mouse samples, n values are indicated in the corresponding figures.

**Metabolic Flux Analysis**

25.000 cells/mL were seeded in 6-well plates and left attach for 48h. Media was then changed to RPMI supplemented with 2 g/L ^13^C-glucose overnight. Cells were scrapped from the plate in ice-cold PBS and centrifuged (1400 rpm 5 min). Intracellular metabolites were extracted from pelleted cells by a double extraction with 100% methanol followed by a single extraction with milli-Q water [20]. The derivatization of intracellular metabolites was performed using BTFSA with 1% TMCS [21]. Finally, 100 µL of hexane were added and transferred into a GC vial for GC-MS analysis. The column temperature was initially held at 60°C for 1 min to 300°C at 10°C /min, held 10 min. Equilibration time was 2 min. Total time of analysis was 37.5 min. Helium was used as a carrier gas at a flow rate of 1 mL/min. The injector temperature was kept at 250°C while the detector transfer line, filament source and quadrupole temperature were set at 280°C, 230°C and 150°C, respectively. A sample volume of 2 µL was injected in splitless mode with 1 min of purge time. The electron ionization source was operated at 70eV. In a GC-QQQ a full fragment cluster for each metabolite was measured in SIM mode using 10 ms of dwell time per mass. The relative contribution of isotope patterns in the experimental mass spectra was calculated by multiple linear regression. Enrichment and pathway analyses were performed using MetaboAnalyst 6.0 [22].

**Proliferation and Cell cycle analysis**

Cells were seeded at 25,000 cells/mL and allowed to attach for 48 hours. For cell counts, cells were collected at specific time points, resuspended in PBS, and mixed 1:1 with 0.4% trypan blue solution to assess cell death. Each experiment was performed in triplicate and repeated three times.

For cell cycle analysis, cells were washed twice with ice-cold PBS containing 1 g/L glucose and fixed in ice-cold 70% ethanol. After overnight fixation at 4°C, cells were centrifuged, resuspended at 106 cells/mL in a solution of 100 µg/mL propidium iodide and 100 µg/mL RNase A in PBS containing 1 g/L glucose, and incubated overnight. PI fluorescence was measured using a Cytoflex S cytometer. For each condition, experiments were performed in triplicate, with at least 10,000 events per replicate analyzed. Data analysis was performed using FlowJo software (BD, v10.9.0). Experiments were repeated three times.

**Apoptosis assay**

Cells were plated at 25,000 cells/mL, allowed to attach for 48 hours, and collected at the given time points following treatments. Cells were centrifuged, counted and resuspended in a working solution of 150 nM YOPRO-1® + 500nM PI in Hank's balanced solution. After 20 minutes of incubation at 37°C, fluorescence was measured using a Cytoflex S cytometer. Three replicates per condition and at least 10,000 events per replicate were analyzed. Experiments were repeated three times, and data analysis was performed using FlowJo software (BD, v10.9.0).

**Colony formation in 2D**

PC-3 cells were seeded at 660 cell/mL. After one week, colonies were visible, and cells were fixed with 4% PFA overnight. After washing with PBS, 0.005% violet crystal was used for 1 hour to stain cells, and then wells were carefully rinsed with water. Plates were scanned and images were analyzed with the GelCount^TM^ Mammalian Cell Colony counter.

**Organoid establishment and culturing conditions**

Organoids from TRAMP-*Txnip*^WT^ and TRAMP-*Txnip*^-/-^ mice were established as previously described [23]. Briefly, 18-week-old mice were euthanized, and the prostate was excised. The tumoral dorsal lobe was separated to undergo mechanical, (scissors cutting) and chemical (5 mg/mL Collagenase type II + Anoikis inhibitor 10 μM Y-27632). The small pieces were then transferred to a 15 mL Falcon tube containing 3 mL digestion mix and were incubated at 37°C for 1.5h. The digested tissue was then turned into a single-cell suspension by incubating the pellet in TryPLE^TM^ with the 10 μM Y-27632 for 5-10 min. TryPLE^TM^ was washed and the cell suspension was counted with trypan blue to seed 20000 viable cells in a 40 μL drop of Growth Factor Reduced Matrigel in a 24-well plate, which was allowed to solidify upside down in the incubator for 30 minutes. Expansion medium was then added to allow the formation and growth of the organoids, whose composition is detailed in Supplementary Table VII. Medium was changed every 3 days, and when the culture reached confluence, a passage was performed removing the medium and adding TryPLE^TM^+Y-27632 to the well for 15 minutes, transferring the content to a clean tube for another 3 min, centrifuging and resuspended until aggregates were removed. All 10 lines successfully grew up to passage 10, showing a high growth rate with 1:10 weekly passages

Supplementary Table I. List of reagents and resources.

| **CELL CULTURE** | | |
| --- | --- | --- |
| CELL LINES | | |
| LNCaP | ATCC (Manasas, VA, USA) | CRL-1740 |
| PC-3 | ATCC | CRL-1435 |
| 293T | ATCC | CRL-11268 |
| PLASMIDS AND VECTORS | | |
| pAX | Addgene (Watertown, MA, USA) | #12260 |
| VSV-G | Addgene | #12259 |
| hTXNIP vector | VectorBuilder (Neu-Isenburg, Germany) | Custom order |
| Empty vector | VectorBuilder | Custom order |
| REAGENTS | | |
| A83-01 | Tocris (Bristol, UK) | 2939/10 |
| AdvancedDMEM/F-12 | Gibco (Waltham, MA USA) | 12634028 |
| B-27 Supplement | Fisher Scientific (Waltham, MA USA) | 17504044 |
| DMEM HG | Merck (Darmstadt, Germany) | D5671 |
| DMEM/F12 | Merck | D6421 |
| DMSO for cell culture | PanReac (Bercelona, Spain) | A3672,0250 |
| FBS | Corning (Corning, NY, USA) | 11573397 |
| Glutamax supplement | Gibco | 35050061 |
| Hank's balanced solution | Merck | H9269 |
| hEGF | Proteintech (Rosemont, IL, USA) | AF-100-15-100UG |
| hNoggin | Proteintech | 120-10C-50UG |
| hR-Spondin | Proteintech | 120-38-250UG |
| L-glutamine | Gibco | 25030081 |
| Low-attachment U-bottom plates | Corning | 4515 |
| Matrigel Growth Factor Reduced | Corning | 356231 |
| Pen/Strep | Gibco | 15140122 |
| Pen/Strep/Amphotericin | Biowest (Bradenton, FL, USA) | L0010-100 |
| Poly-D-lysine | Merck | A-003-E |
| Puromycin | GoldBio (St Louis, MO, USA) | P-600-100 |
| RPMI-1640 | Merck | R0883 |
| Seahorse Calibrant | Agilent (Santa Clara, CA, USA) | 100840 |
| Seahorse glucose | Agilent | 103577 |
| Seahorse pyruvate | Agilent | 103578-100 |
| Seahorse RPMI | Agilent | 103576-100 |
| Seahorse XFp Miniplates | Agilent | 103025-100 |
| Trypan Blue solution | Amresco (Solon, OH, USA) | 1-800-448-4442 |
| TryPLE Express | Gibco | 12605010 |
| Trypsin-EDTA 0.05% | Fisher Scientific | 25300-062 |
| Trypsin-EDTA 0.25% | Gibco | 25200-072 |
| Y-27632 | MedChem (Monmouth Junction, NJ, USA) | HY-10583-10MG |
| **EXPERIMENTAL MODELS: MICE** |  |  |
| C57BL/6-Tg(TRAMP)8247Ng/J | The Jackson Laboratory | 003135 |
| B6;129-Txnip^tm1Rlee^/J | The Jackson Laboratory | 016847 |
| **KITS** | | |
| CellTiter^TM^ Glo | Promega (Madison, WI, USA) | G9681 |
| Histogel (Gel Specimen Processing) | Epredia (Portsmouth, NH, USA) | HG-4000-012 |
| L-lactate Assay kit | Merck | MAK329-1KT |
| Mycoplasma detection kit | Biotools (Jupiter, FL, USA) | 90-021 |
| β-galactosidase staining kit | Cell Signaling (Danvers, MA, USA) | 9860 |
| Simple ChIP kit (Magnetic beads) | Cell Signaling | 9003 |
| Testosterone ELISA kit | Cayman Chemical (Ann Arbor, MI, USA) | 582701 |
| **DRUGS** | | |
| 2-Deoxyglucose | Alfa Aesar (Haverhill, MA, USA) | L07338 |
| Antimycin A | Merck | A8674 |
| Casodex | Merck | B9061 |
| DHT | Merck | A8505 |
| FCCP | Merck | C2920 |
| Oligomycin | Merck | O4876 |
| Rotenone | Merck | R8875 |
| SRI-37330 | MedChem | HY-141623 |
| **PROBES** | | |
| CellROX | Invitrogen (Waltham, MA USA) | C10422 |
| DHE | Invitrogen | D11347 |
| JC-1 | Invitrogen | T3168 |
| MitoGreen | Invitrogen | M7514 |
| MitoSOX | Invitrogen | M36008 |
| Propidium iodide | Merck | 81845 |
| YO-PRO1 | Invitrogen | Y3603 |
| **ANTIBODIES** | | |
| Alexa Fluor 488 anti-rabbit | Invitrogen | A11008 |
| Alexa Fluor 488 anti-rat | Invitrogen | A11006 |
| Alexa Fluor 594 anti-rabbit | Invitrogen | A11012 |
| Anti-Actin | SantaCruz (Dallas, TX, USA) | sc69879 |
| Anti-AR | Cell Signaling | 5153S |
| Anti-Catalase | Calbiochem (San Diego, CA, USA) | 219010 |
| Anti-CD3 | Abcam | ab135372 |
| Anti-CK5 | Abcam (Cambridge, UK) | ab52635 |
| Anti-CK8 | Merck | MABT329M |
| Anti-Cyclin A | SantaCruz | sc-271682 |
| Anti-GAPDH | SantaCruz | sc-166545 |
| Anti-GLUT1 | Merck | 07-1401 |
| Anti-GPX4 | Cell signaling | 59735SX |
| Anti-HDAC | Santa Cruz | sc9959 |
| Anti-Ki67 | Roche (Basel, Switzerland) | 5278384001 |
| Anti-mouse HRP-conjugated | Merck | 12-349 |
| Anti-mouse IRDye 680RD | LI-COR (Lincoln, NE, USA) | 92668070 |
| Anti-p27^kip1^ | Cell signaling | 3698 |
| Anti-pRb Ser780 | Cell signaling | 9307 |
| Anti-PRDX6 | Invitrogen | PA5-96040 |
| Anti-rabbit HRP-conjugated | Invitrogen | 31466 |
| Anti-Rabbit IRDye 800RD | LI-COR | 92632211 |
| Anti-Rb | Cell signaling | 9309 |
| Anti-SOD1 | Calbiochem | 574596 |
| Anti-SOD2 | Merck | 06-984 |
| Anti-TRX1 | Cell signaling | 2429S |
| Anti-TRX2 | Cell signaling | 14907 |
| Anti-TXNIP | Abcam | ab188865 |
| **OTHER REAGENTS** | | |
| BSA | Merck | A9647 |
| Bupaq | Richter Pharma (Wels, Austria) | 383/01/11DFVPT |
| CaCl_2_ | Merck | A863082 |
| Charcoal activated | Merck | 1.02186.0250 |
| Collagenase Type II | Gibco | 17101015 |
| Complete^TM^ Protease inhibitor cocktail | Roche | 11697498001 |
| DAPI | VWR (Radnor, PA, USA) | A4099.0010 |
| Dispase II | Merck | D4693-1G |
| DTT | Alfa Aesar | J64545 |
| ECL | Merck | WBULS0100 |
| Glycerol | Merck | 102416780 |
| HEPES | Thermo Scientific | A14777.30 |
| Igepal | Merck | I3021 |
| KCl | Alfa Aesar | 11595 |
| Metacam | Boehringer Ingelheim (Ingelheim, Germany) | 059/02/08CVPT |
| MgCl_2_ | Fluka | 63068 |
| NaCl | VWR | 27810 |
| NaF | PanReac | A3904,0025 |
| NZYol | Nzytech (Lisboa, Portugal) | MB18501 |
| PVDF membranes | Merck | IPFL00005 |
| RNAse A | Merck | R4642 |
| First-strand cDNA Synthesis | Nzytech | MB40001 |
| SDS | Thermo Scientific | A11183.22 |
| Sodium deoxycolate | Merck | D6750 |
| Sodium orthovanadate | Merck | S6508 |
| qPCR Green Master Mix | Nzytech | MB44003 |
| Tris | Fisher Scientific | BP152-1 |
| **SOFTWARE** |  |  |
| Agilent Seahorse Analytics | https://www.agilent.com | |
| Biorender | https://www.biorender.com | |
| FlowJo 10.9 | https://www.flowjo.com | |
| GraphPad Prism 8.0.2 | https://www.graphpad.com | |
| MetaboAnalyst 6.0 | https://www.metaboanalyst.ca | |

Supplementary Table II. Clinical data from patients enrolled in the study.

Supplementary Table III. Antibody clone ID and concentration

| **ANTIBODY** | **Clone ID** | **USE** | **DILUTION** |
| --- | --- | --- | --- |
| Anti-Actin | AC-15 | WB | 1:15000 |
| Anti-Catalase | - | WB | 1:3000 |
| Anti-Cyclin A | B-8 | WB | 1:1000 |
| Anti-GAPDH | D-6 | WB | 1:1000 |
| Anti-GLUT1 | - | IF | 1:200 |
| Anti-GPX4 | E5Y8K | WB | 1:1000 |
| Anti-HDAC | C-8 | WB | 1:500 |
| Anti-Ki67 | 30-9 | IHC | Ready to use (2 μg/mL) |
| Anti-p27^kip1^ | SX5368.5 | WB | 1:1000 |
| Anti-pRb Ser780 | - | WB | 1:2000 |
| Anti-PRDX6 | - | WB | 1:500 |
| Anti-Rb | 4H1 | WB | 1:1000 |
| Anti-SOD1 | - | WB | 1:2000 |
| Anti-SOD2 | - | WB | 1:4000 |
| Anti-TRX1 | C63C6 | WB | 1:2000 |
| Anti-TRX2 | D1C9L | WB | 1:1000 |
| Anti-TXNIP | EPR14774 | WB | 1:2000 |
| Anti-TXNIP | EPR14774 | IF | 1:200 |

Supplementary Table IV. IF conditions.

Supplementary Table V. List of primers and their sequences used in the present work.

| **PRIMERS** | | |
| --- | --- | --- |
| GENOTYPING | | |
| TG1 F | Celta Ingenieros | 5'-AGGCATTCCACCACTGCTCCCATTCATC-3' |
| TG1 R | Celta Ingenieros | 5'-CCGGTCCACCGGAAGCTTCCACAAGTGCATTTA-3' |
| TG2 F | Celta Ingenieros | 5'-GCGCTGCTGACTTTCTAAACATAAG-3' |
| TG2 R | Celta Ingenieros | 5'-GAGCTCAGCTTAAGTTTTGATGTGT-3' |
| Mutant Txnip | Celta Ingenieros | 5'-TTTCGTTTGCGTTTTCAAGC-3' |
| Common Txnip | Celta Ingenieros | 5'-CCCAGAGCAGTTTCTTGGAC-3' |
| WT Txnip | Celta Ingenieros | 5'-CTTCACCCCCCTAGAGTGAT-3' |
| qPCR | | |
| *ACTB* F | Celta Ingenieros | 5'-GGCTGTATTCCCCTCCATCG-3' |
| *ACTB* R | Celta Ingenieros | 5'-CCAGTTGGTAACAATGCCATGT-3' |
| *Actb* F | Celta Ingenieros | 5'-AAGATCAAGATCATTGCTCCTCC-3' |
| *Actb* R | Celta Ingenieros | 5'-GTCATAGTCCGCCTAGAAGCA-3' |
| *Fkbp5* F | Merck | 5’-TGAGGGCACCAGTAACAATGG-3' |
| *Fkbp5* R | Merck | 5’-CAACATCCCTTTGTAGTGGACAT-3' |
| *TXNIP* F | Celta Ingenieros | 5'-TCTTTTGAGGTGGTCTTCAACG-3' |
| *TXNIP* R | Celta Ingenieros | 5'-GCTTTGACTCGGGTAACTTCACA-3' |
| *TXNIP promoter F* | Celta Ingenieros | 5’- TCCAGAGCGCAACAACCAT-3’ |
| *TXNIP promoter R* | Celta Ingenieros | 5’- AAGCAGGAGGCGGAAACGT-3’ |
| *Txnip* F | Celta Ingenieros | 5'-CTTACTGATCTATGTTAGCGTTCC-3' |
| *Txnip* R | Celta Ingenieros | 5'-GGATGTTCAGATCTACCCAACT-3' |

Supplementary Table VI. Organoid medium composition (Drost et al, 2016).

| **ORGANOID MEDIUM** | |
| --- | --- |
| BASAL MEDIUM | |
| AdDMEM/F12 | - |
| GlutaMAX | 2 mM |
| Pen/Strep | 1% |
| HEPES | 10 mM |
| GROWTH FACTORS | |
| B27 | 1x |
| NAC | 1.25 mM |
| R-SPONDIN1 | 500 ng/mL |
| NOGGIN | 100 ng/mL |
| EGF | 50 ng/mL |
| A83-01 | 200 nM |
| GROWTH FACTORS - OPTIONAL | |
| DHT | 1 nM |
| Y-27632 | 10 μM |

| Supplementary Table VII. Patient clinical data from cohorts used in Fig. 1.  **Liu *et al*, 2016 (10.1158/0008-5472.CAN-05-3055)** | | | | | |
| --- | --- | --- | --- | --- | --- |
| **Sample ID** | **Sample type** | **Age (years)** | **Gleason Score** | **Disease staging** | *TXNIP* expression |
| MBA: A-185N | Normal prostate | 59 | NA | PT2b | 0.649 |
| MBA: A-399N | Normal prostate | 55 | NA | PT2b | 0.107 |
| MBA: A-340N | Normal prostate | 50 | NA | PT2b | -0.160 |
| MBA: A-V16N | Normal prostate | 59 | NA | PT2 | -0.307 |
| MBA: A-EP04N | Normal prostate | 66 | NA | PT2b | -0.172 |
| MBA: A-V21N | Normal prostate | 64 | NA | PT2 | 0.084 |
| MBA: A-V19N | Normal prostate | 57 | NA | PT2 | -0.199 |
| MBA: A-EP03N | Normal prostate | 54 | NA | PT2b | 0.150 |
| MBA: A-23N | Normal prostate | 65 | NA | PT3 | -0.847 |
| MBA: A-EP02N | Normal prostate | 63 | NA | PT2b | 0.032 |
| MBA: A-V29N | Normal prostate | 72 | NA | PT3 | 0.154 |
| MBA: A-EP06N | Normal prostate | 60 | NA | PT2b | -0.235 |
| MBA: A-EP01N | Normal prostate | 55 | NA | PT2b | 0.241 |
| MBA: 340T | prostate cancer | 50 | Gleason 7 | PT2b | -0.745 |
| MBA: 362T | prostate cancer | 58 | Gleason 6 | PT2b | -1.297 |
| MBA: A-4418592762 | prostate cancer | 66 | Gleason 8 | PT2b | 2.416 |
| MBA: A-257T | prostate cancer | 56 | Gleason 7 | PT2b | -2.511 |
| MBA: 195T-A | prostate cancer | 55 | Gleason 7 | PT3a | -0.433 |
| MBA: A-EP06T | prostate cancer | 60 | Gleason 6 | PT2b | -0.333 |
| MBA: A-EP01T | prostate cancer | 55 | Gleason 6 | PT2b | -0.825 |
| MBA: 246T-A | prostate cancer | 66 | Gleason 8 | PT4a | -0.774 |
| MBA: A-V30T | prostate cancer | 56 | Gleason 7 | PT3 | -0.667 |
| MBA: A-2393346053 | prostate cancer | 53 | Gleason 8 | PT2b | -0.845 |
| MBA: A-3010184133 | prostate cancer | 60 | Gleason 6 | PT2b | -0.849 |
| MBA: A-4917290232 | prostate cancer | 61 | Gleason 8 | PT3b | -1.189 |
| MBA: A-5642567629 | prostate cancer | 48 | Gleason 8 | PT2a | -1.525 |
| MBA: 111T-A | prostate cancer | 59 | Gleason 7 | PT2a | -0.119 |
| MBA: A-226T | prostate cancer | 50 | Gleason 6 | PT2b | -1.091 |
| MBA: 399T | prostate cancer | 55 | Gleason 6 | PT2b | 0.216 |
| MBA: A-237T | prostate cancer | 55 | Gleason 8+ | PT2b | -0.492 |
| MBA: A-5173529673 | prostate cancer | 66 | Gleason 8 | PT3b | 0.279 |
| MBA: A-EP04T | prostate cancer | 66 | Gleason 7 | PT2b | -1.369 |
| MBA: 405T | prostate cancer | 55 | Gleason 6 | NA | -1.022 |
| MBA: 357T | prostate cancer | 56 | Gleason 7 | PT2b | 0.119 |
| MBA: A-V21T | prostate cancer | 64 | Gleason 7 | PT2 | -0.381 |
| MBA: A-1940339465 | prostate cancer | 64 | Gleason 8+ | PT3a | 0.386 |
| MBA: 370T | prostate cancer | 58 | Gleason 7 | PT2b | -0.434 |
| MBA: A-4464625690 | prostate cancer | 60 | Gleason 6 | PT3a | -1.043 |
| MBA: A-23T | prostate cancer | 65 | Gleason 8 | PT3 | -0.648 |
| MBA: A-EP03T | prostate cancer | 54 | Gleason 6 | PT2b | -0.488 |
| MBA: A-7270793196 | prostate cancer | 63 | Gleason 8 | PT2b | -0.287 |
| MBA: A-3435720971 | prostate cancer | 62 | Gleason 8 | PT3a | -0.251 |
| MBA: A-7350218006 | prostate cancer | 62 | Gleason 6 | PT2a | -0.231 |
| MBA: A-5292628126 | prostate cancer | 55 | Gleason 8+ | PT3b | -0.907 |
| MBA: A-135T | prostate cancer | 72 | Gleason 7 | PT2b | -1.155 |
| MBA: A-8500920543 | prostate cancer | 58 | Gleason 8+ | PT3a | -0.224 |
| MBA: A-4963842013 | prostate cancer | 52 | Gleason 8+ | PT3a | 0.174 |
| MBA: A-EP02T | prostate cancer | 63 | Gleason 6 | PT2b | -0.821 |
| MBA: A-V29T | prostate cancer | 72 | Gleason 6 | PT3 | -0.449 |
| MBA: A-4472570235 | prostate cancer | 62 | Gleason 8 | PT3a | -0.282 |
| MBA: 243T-A | prostate cancer | 64 | Gleason 7 | PT3b | -0.031 |
| MBA: A-V16T | prostate cancer | 59 | Gleason 7 | PT2 | -0.678 |
| MBA: A-V19T | prostate cancer | 57 | Gleason 7 | PT2 | -0.206 |
| MBA: A-9763059872 | prostate cancer | 60 | Gleason 6 | PT3a | 0.662 |
| MBA: A-169T | prostate cancer | 43 | Gleason 7 | PT3 | -1.182 |
| MBA: A-171T | prostate cancer | 66 | Gleason 7 | PT3b | 0.115 |
| MBA: 185T-A | prostate cancer | 59 | Gleason 7 | PT2b | -0.219 |

| **Mortensen *et al*, 2015 (10.1038/srep16018)** | | | | | | | | |
| --- | --- | --- | --- | --- | --- | --- | --- | --- |
| **Sample ID** | **Sample type** | **Age (years)** | **Gleason Score** | **Disease staging** | **Preoperatory PSA** | **Biochemical recurrence** | **Time to BCR** | *TXNIP* expression |
| GSM1133150 | benign prostate glands | 62 | NA | NA | NA | NA | NA | 1.150 |
| GSM1133151 | benign prostate glands | 68 | NA | NA | NA | NA | NA | 1.068 |
| GSM1133163 | benign prostate glands | 58 | NA | NA | NA | NA | NA | 1.157 |
| GSM1133166 | benign prostate glands | 56 | NA | NA | NA | NA | NA | 1.182 |
| GSM1133177 | benign prostate glands | 75 | NA | NA | NA | NA | NA | 1.257 |
| GSM1133179 | benign prostate glands | 53 | NA | NA | NA | NA | NA | 1.201 |
| GSM1133180 | benign prostate glands | 74 | NA | NA | NA | NA | NA | 1.249 |
| GSM1133181 | benign prostate glands | 62 | NA | NA | NA | NA | NA | 1.163 |
| GSM1133183 | benign prostate glands | 70 | NA | NA | NA | NA | NA | 1.254 |
| GSM1133184 | benign prostate glands | 52 | NA | NA | NA | NA | NA | 1.283 |
| GSM1133185 | benign prostate glands | 69 | NA | NA | NA | NA | NA | 1.243 |
| GSM1133136 | prostate tumor | 57 | Gleason 5 | pT3b | 11.4 | YES | 9 | 1.090 |
| GSM1133137 | prostate tumor | 60 | Gleason 6 | pT3a | 19.8 | YES | 7 | 1.175 |
| GSM1133138 | prostate tumor | 62 | Gleason 6 | pT2c | 10.5 | NO | 80.63 | 1.114 |
| GSM1133140 | prostate tumor | 63 | Gleason 6 | pT3a | 23 | YES | 5 | 1.095 |
| GSM1133141 | prostate tumor | 58 | Gleason 7 | pT3a | 9.1 | YES | 64 | 1.135 |
| GSM1133142 | prostate tumor | 57 | Gleason 5 | pT2c | 6.7 | NO | 79.43 | 1.138 |
| GSM1133143 | prostate tumor | 68 | Gleason 8 | pT2c | 13.2 | YES | 70 | 1.109 |
| GSM1133144 | prostate tumor | 68 | Gleason 7 | pT2c | 10.5 | YES | 16 | 1.131 |
| GSM1133145 | prostate tumor | 59 | Gleason 5 | pT3a | 25.7 | YES | 15 | 1.217 |
| GSM1133146 | prostate tumor | 67 | Gleason 6 | pT2c | 21.2 | NO | 77.6 | 1.149 |
| GSM1133147 | prostate tumor | 68 | Gleason 4 | pT2a | 10.3 | NO | 77.37 | 1.145 |
| GSM1133148 | prostate tumor | 59 | Gleason 7 | pT3a | 10.7 | YES | 40 | 1.109 |
| GSM1133149 | prostate tumor | 69 | Gleason 6 | pT2c | 15.5 | NO | 76.17 | 1.118 |
| GSM1133152 | prostate tumor | 65 | Gleason 5 | pT2a | 7.9 | NO | 74.53 | 1.203 |
| GSM1133153 | prostate tumor | 61 | Gleason 5 | pT2c | 8.6 | NO | 74.1 | 1.003 |
| GSM1133154 | prostate tumor | 58 | Gleason 7 | pT3a | 25.5 | YES | 74.7 | 1.136 |
| GSM1133155 | prostate tumor | 59 | Gleason 6 | pT2c | 11.8 | YES | 49 | 1.160 |
| GSM1133157 | prostate tumor | 71 | Gleason 7 | pT3b | 20.1 | YES | 15 | 1.225 |
| GSM1133158 | prostate tumor | 55 | Gleason 6 | pT2a | 22.2 | NO | 58.6 | 1.248 |
| GSM1133160 | prostate tumor | 53 | Gleason 5 | pT3a | 9.9 | NO | 54.63 | 1.145 |
| GSM1133161 | prostate tumor | 59 | Gleason 7 | pT3b | 32.2 | YES | 31 | 0.884 |
| GSM1133164 | prostate tumor | 67 | Gleason 5 | pT2c | 5.3 | NO | 42.07 | 1.166 |
| GSM1133165 | prostate tumor | 68 | Gleason 7 | pT3b | 42.5 | YES | 22 | 1.160 |
| GSM1133167 | prostate tumor | 63 | Gleason 6 | pT2c | 16.5 | NO | 40.87 | 1.243 |
| GSM1133168 | prostate tumor | 46 | Gleason 7 | pT2c | 23.5 | YES | 4 | 1.223 |
| GSM1133170 | prostate tumor | 64 | Gleason 6 | pT2c | 16.6 | NO | 35.8 | 1.199 |
| GSM1133171 | prostate tumor | 63 | Gleason 8 | pT2c | 32.4 | YES | 1 | 1.155 |
| GSM1133172 | prostate tumor | 58 | Gleason 7 | pT2c | 9.9 | NO | 32.77 | 1.094 |
| GSM1133173 | prostate tumor | 57 | Gleason 9 | pT2c | 7.1 | NO | 30.93 | 1.038 |
| GSM1133174 | prostate tumor | 67 | Gleason 7 | pT3b | 7.7 | YES | 31 | 1.132 |
| GSM1133175 | prostate tumor | 63 | Gleason 8 | pT3b | 12.9 | YES | 6 | 1.219 |

| **Grasso *et al*, 2012 (10.1038/nature11125)** | | | | | | |
| --- | --- | --- | --- | --- | --- | --- |
| **Sample Accession** | **Sample type** | **Age (years)** | **Gleason grade** | **Disease staging** | **Prostate cancer** | *TXNIP* expression |
| GSM878766 | Normal prostate | N.A. | N.A. | N.A. | normal prostate | -1.232 |
| GSM878767 | Normal prostate | N.A. | N.A. | N.A. | normal prostate | -0.633 |
| GSM878768 | Normal prostate | N.A. | N.A. | N.A. | normal prostate | -1.200 |
| GSM878769 | Normal prostate | N.A. | N.A. | N.A. | normal prostate | -0.765 |
| GSM878770 | Normal prostate | N.A. | N.A. | N.A. | normal prostate | -0.892 |
| GSM878771 | Normal prostate | N.A. | N.A. | N.A. | normal prostate | -0.108 |
| GSM878772 | Normal prostate | N.A. | N.A. | N.A. | normal prostate | -1.016 |
| GSM878773 | Normal prostate | N.A. | N.A. | N.A. | normal prostate | -0.514 |
| GSM878774 | Normal prostate | N.A. | N.A. | N.A. | normal prostate | -0.369 |
| GSM878775 | Normal prostate | N.A. | N.A. | N.A. | normal prostate | -1.537 |
| GSM878776 | Normal prostate | N.A. | N.A. | N.A. | normal prostate | -1.190 |
| GSM878777 | Normal prostate | N.A. | N.A. | N.A. | normal prostate | -1.005 |
| GSM878750 | Normal prostate | N.A. | N.A. | N.A. | normal prostate | -1.153 |
| GSM878751 | Normal prostate | N.A. | N.A. | N.A. | normal prostate | -1.250 |
| GSM878752 | Normal prostate | N.A. | N.A. | N.A. | normal prostate | -0.159 |
| GSM878753 | Normal prostate | N.A. | N.A. | N.A. | normal prostate | -1.665 |
| GSM878754 | Normal prostate | N.A. | N.A. | N.A. | normal prostate | -1.152 |
| GSM878755 | Normal prostate | N.A. | N.A. | N.A. | normal prostate | -1.478 |
| GSM878756 | Normal prostate | N.A. | N.A. | N.A. | normal prostate | -1.020 |
| GSM878757 | Normal prostate | N.A. | N.A. | N.A. | normal prostate | -1.056 |
| GSM878758 | Normal prostate | N.A. | N.A. | N.A. | normal prostate | -1.174 |
| GSM878759 | Normal prostate | N.A. | N.A. | N.A. | normal prostate | -1.417 |
| GSM878760 | Normal prostate | N.A. | N.A. | N.A. | normal prostate | -1.303 |
| GSM878761 | Normal prostate | N.A. | N.A. | N.A. | normal prostate | -2.099 |
| GSM878762 | Normal prostate | N.A. | N.A. | N.A. | normal prostate | -0.187 |
| GSM878763 | Normal prostate | N.A. | N.A. | N.A. | normal prostate | -1.663 |
| GSM878764 | Normal prostate | N.A. | N.A. | N.A. | normal prostate | -1.542 |
| GSM878765 | Normal prostate | N.A. | N.A. | N.A. | normal prostate | -0.202 |
| GSM878788 | Prostate cancer | N.A. | N.A. | N.A. | localized Pca | -1.325 |
| GSM878789 | Prostate cancer | N.A. | N.A. | N.A. | localized Pca | -1.371 |
| GSM878790 | Prostate cancer | N.A. | N.A. | N.A. | localized Pca | -1.106 |
| GSM878791 | Prostate cancer | N.A. | N.A. | N.A. | localized Pca | -1.168 |
| GSM878792 | Prostate cancer | N.A. | N.A. | N.A. | localized Pca | -0.947 |
| GSM878793 | Prostate cancer | N.A. | N.A. | N.A. | localized Pca | -1.343 |
| GSM878794 | Prostate cancer | N.A. | N.A. | N.A. | localized Pca | -1.316 |
| GSM878795 | Prostate cancer | N.A. | N.A. | N.A. | localized Pca | -1.555 |
| GSM878796 | Prostate cancer | N.A. | N.A. | N.A. | localized Pca | -1.128 |
| GSM878797 | Sample type | N.A. | N.A. | N.A. | localized Pca | -1.417 |
| GSM878798 | Prostate cancer | N.A. | N.A. | N.A. | localized Pca | -0.880 |
| GSM878799 | Prostate cancer | N.A. | N.A. | N.A. | localized Pca | -1.417 |
| GSM878800 | Prostate cancer | N.A. | N.A. | N.A. | localized Pca | -0.669 |
| GSM878801 | Prostate cancer | N.A. | N.A. | N.A. | localized Pca | -1.200 |
| GSM878802 | Prostate cancer | N.A. | N.A. | N.A. | localized Pca | -1.876 |
| GSM878803 | Prostate cancer | N.A. | N.A. | N.A. | localized Pca | -2.316 |
| GSM878804 | Prostate cancer | N.A. | N.A. | N.A. | localized Pca | -1.158 |
| GSM878805 | Prostate cancer | N.A. | N.A. | N.A. | localized Pca | -1.827 |
| GSM878806 | Prostate cancer | N.A. | N.A. | N.A. | localized Pca | -1.188 |
| GSM878807 | Prostate cancer | N.A. | N.A. | N.A. | localized Pca | -2.051 |
| GSM878808 | Prostate cancer | N.A. | N.A. | N.A. | localized Pca | -1.227 |
| GSM878809 | Prostate cancer | N.A. | N.A. | N.A. | localized Pca | -1.766 |
| GSM878810 | Prostate cancer | N.A. | N.A. | N.A. | localized Pca | -1.765 |
| GSM878811 | Prostate cancer | N.A. | N.A. | N.A. | localized Pca | -1.152 |
| GSM878812 | Prostate cancer | N.A. | N.A. | N.A. | localized Pca | -3.169 |
| GSM878813 | Prostate cancer | N.A. | N.A. | N.A. | localized Pca | -1.303 |
| GSM878814 | Prostate cancer | N.A. | N.A. | N.A. | localized Pca | -2.116 |
| GSM878815 | Prostate cancer | N.A. | N.A. | N.A. | localized Pca | -1.742 |
| GSM878816 | Prostate cancer | N.A. | N.A. | N.A. | localized Pca | -1.429 |
| GSM878817 | Prostate cancer | N.A. | N.A. | N.A. | localized Pca | -1.951 |
| GSM878818 | Prostate cancer | N.A. | N.A. | N.A. | localized Pca | -1.476 |
| GSM878819 | Prostate cancer | N.A. | N.A. | N.A. | localized Pca | -1.521 |
| GSM878820 | Prostate cancer | N.A. | N.A. | N.A. | localized Pca | -1.022 |
| GSM878821 | Prostate cancer | N.A. | N.A. | N.A. | localized Pca | -1.441 |
| GSM878822 | Prostate cancer | N.A. | N.A. | N.A. | localized Pca | -1.344 |
| GSM878823 | Prostate cancer | N.A. | N.A. | N.A. | localized Pca | -1.006 |
| GSM878824 | Prostate cancer | N.A. | N.A. | N.A. | localized Pca | -1.324 |
| GSM878825 | Prostate cancer | N.A. | N.A. | N.A. | localized Pca | -1.097 |
| GSM878826 | Prostate cancer | N.A. | N.A. | N.A. | localized Pca | -1.589 |
| GSM878827 | Prostate cancer | N.A. | N.A. | N.A. | localized Pca | -2.663 |
| GSM878828 | Prostate cancer | N.A. | N.A. | N.A. | localized Pca | -1.159 |
| GSM878829 | Prostate cancer | N.A. | N.A. | N.A. | localized Pca | -3.152 |
| GSM878830 | Prostate cancer | N.A. | N.A. | N.A. | localized Pca | -4.042 |
| GSM878831 | Prostate cancer | N.A. | N.A. | N.A. | localized Pca | -1.015 |
| GSM878832 | Prostate cancer | N.A. | N.A. | N.A. | localized Pca | -0.029 |
| GSM878833 | Prostate cancer | N.A. | N.A. | N.A. | localized Pca | -0.208 |
| GSM878834 | Prostate cancer | N.A. | N.A. | N.A. | localized Pca | -2.605 |
| GSM878835 | Prostate cancer | N.A. | N.A. | N.A. | localized Pca | -2.110 |
| GSM878836 | Prostate cancer | N.A. | N.A. | N.A. | localized Pca | -0.203 |
| GSM878778 | Prostate cancer | N.A. | N.A. | N.A. | localized Pca | -0.382 |
| GSM878779 | Prostate cancer | N.A. | N.A. | N.A. | localized Pca | -2.099 |
| GSM878780 | Prostate cancer | N.A. | N.A. | N.A. | localized Pca | -0.187 |
| GSM878781 | Prostate cancer | N.A. | N.A. | N.A. | localized Pca | -1.663 |
| GSM878782 | Prostate cancer | N.A. | N.A. | N.A. | localized Pca | -1.542 |
| GSM878783 | Prostate cancer | N.A. | N.A. | N.A. | localized Pca | -0.906 |
| GSM878784 | Prostate cancer | N.A. | N.A. | N.A. | localized Pca | -2.183 |
| GSM878785 | Prostate cancer | N.A. | N.A. | N.A. | localized Pca | -0.140 |
| GSM878786 | Prostate cancer | N.A. | N.A. | N.A. | localized Pca | -0.761 |
| GSM878787 | Prostate cancer | N.A. | N.A. | N.A. | localized Pca | -1.657 |
| GSM878845 | Prostate cancer | N.A. | N.A. | N.A. | mCRPC | -3.114 |
| GSM878846 | Prostate cancer | N.A. | N.A. | N.A. | mCRPC | -2.839 |
| GSM878847 | Prostate cancer | N.A. | N.A. | N.A. | mCRPC | -2.123 |
| GSM878848 | Prostate cancer | N.A. | N.A. | N.A. | mCRPC | -2.320 |
| GSM878849 | Prostate cancer | N.A. | N.A. | N.A. | mCRPC | -2.389 |
| GSM878850 | Prostate cancer | N.A. | N.A. | N.A. | mCRPC | -1.998 |
| GSM878851 | Prostate cancer | N.A. | N.A. | N.A. | mCRPC | -2.102 |
| GSM878852 | Prostate cancer | N.A. | N.A. | N.A. | mCRPC | -1.194 |
| GSM878853 | Prostate cancer | N.A. | N.A. | N.A. | mCRPC | -1.918 |
| GSM878854 | Prostate cancer | N.A. | N.A. | N.A. | mCRPC | -1.338 |
| GSM878855 | Prostate cancer | N.A. | N.A. | N.A. | mCRPC | -2.110 |
| GSM878856 | Prostate cancer | N.A. | N.A. | N.A. | mCRPC | -0.203 |
| GSM878857 | Prostate cancer | N.A. | N.A. | N.A. | mCRPC | -3.114 |
| GSM878858 | Prostate cancer | N.A. | N.A. | N.A. | mCRPC | -2.839 |
| GSM878859 | Prostate cancer | N.A. | N.A. | N.A. | mCRPC | -2.123 |
| GSM878860 | Prostate cancer | N.A. | N.A. | N.A. | mCRPC | -2.320 |
| GSM878861 | Prostate cancer | N.A. | N.A. | N.A. | mCRPC | -0.243 |
| GSM878862 | Prostate cancer | N.A. | N.A. | N.A. | mCRPC | -2.389 |
| GSM878863 | Prostate cancer | N.A. | N.A. | N.A. | mCRPC | 0.771 |
| GSM878864 | Prostate cancer | N.A. | N.A. | N.A. | mCRPC | 0.561 |
| GSM878865 | Prostate cancer | N.A. | N.A. | N.A. | mCRPC | -1.998 |
| GSM878866 | Prostate cancer | N.A. | N.A. | N.A. | mCRPC | -0.507 |
| GSM878867 | Prostate cancer | N.A. | N.A. | N.A. | mCRPC | -0.020 |
| GSM878868 | Prostate cancer | N.A. | N.A. | N.A. | mCRPC | -2.102 |
| GSM878869 | Prostate cancer | N.A. | N.A. | N.A. | mCRPC | -1.194 |
| GSM878870 | Prostate cancer | N.A. | N.A. | N.A. | mCRPC | -1.918 |
| GSM878871 | Prostate cancer | N.A. | N.A. | N.A. | mCRPC | -1.338 |
| GSM878837 | Prostate cancer | N.A. | N.A. | N.A. | mCRPC | -1.576 |
| GSM878838 | Prostate cancer | N.A. | N.A. | N.A. | mCRPC | -2.019 |
| GSM878839 | Prostate cancer | N.A. | N.A. | N.A. | mCRPC | -4.092 |
| GSM878840 | Prostate cancer | N.A. | N.A. | N.A. | mCRPC | -0.205 |
| GSM878841 | Prostate cancer | N.A. | N.A. | N.A. | mCRPC | -3.604 |
| GSM878842 | Prostate cancer | N.A. | N.A. | N.A. | mCRPC | -1.258 |
| GSM878843 | Prostate cancer | N.A. | N.A. | N.A. | mCRPC | -2.072 |
| GSM878844 | Prostate cancer | N.A. | N.A. | N.A. | mCRPC | -1.574 |

| **Yun *et al*, 2017 (10.18632/oncotarget.22296)** | | | | | | |
| --- | --- | --- | --- | --- | --- | --- |
| **Sample Accession** | **Sample type** | **Age (years)** | **Gleason grade** | **Disease staging** | **Prostate cancer** | *TXNIP* expression |
| GSM2131564 | nomal prostate | N.A. | N.A. | N.A. | N.A. | 8.707 |
| GSM2131565 | nomal prostate | N.A. | N.A. | N.A. | N.A. | 8.680 |
| GSM2131566 | nomal prostate | N.A. | N.A. | N.A. | N.A. | 8.799 |
| GSM2131567 | nomal prostate | N.A. | N.A. | N.A. | N.A. | 8.577 |
| GSM2131568 | nomal prostate | N.A. | N.A. | N.A. | N.A. | 8.500 |
| GSM2131569 | nomal prostate | N.A. | N.A. | N.A. | N.A. | 8.494 |
| GSM2131570 | nomal prostate | N.A. | N.A. | N.A. | N.A. | 8.443 |
| GSM2131571 | nomal prostate | N.A. | N.A. | N.A. | N.A. | 8.670 |
| GSM2131572 | prostate cancer | N.A. | N.A. | N.A. | CaP | 6.962 |
| GSM2131573 | prostate cancer | N.A. | N.A. | N.A. | CaP | 5.871 |
| GSM2131574 | prostate cancer | N.A. | N.A. | N.A. | advanced CaP | 7.743 |
| GSM2131575 | prostate cancer | N.A. | N.A. | N.A. | CRPC | 7.733 |
| GSM2131576 | prostate cancer | N.A. | N.A. | N.A. | CaP | 7.582 |
| GSM2131577 | prostate cancer | N.A. | N.A. | N.A. | CRPC | 7.001 |
| GSM2131578 | prostate cancer | N.A. | N.A. | N.A. | CaP | 7.886 |
| GSM2131579 | prostate cancer | N.A. | N.A. | N.A. | CRPC | 8.152 |
| GSM2131580 | prostate cancer | N.A. | N.A. | N.A. | CRPC | 8.131 |
| GSM2131581 | prostate cancer | N.A. | N.A. | N.A. | advanced CaP | 7.370 |
| GSM2131582 | prostate cancer | N.A. | N.A. | N.A. | CaP | 7.649 |
| GSM2131583 | prostate cancer | N.A. | N.A. | N.A. | CaP | 7.505 |
| GSM2131584 | prostate cancer | N.A. | N.A. | N.A. | CRPC | 6.103 |
| GSM2131585 | prostate cancer | N.A. | N.A. | N.A. | CaP | 6.475 |
| GSM2131586 | prostate cancer | N.A. | N.A. | N.A. | CaP | 7.219 |
| GSM2131587 | prostate cancer | N.A. | N.A. | N.A. | CRPC | 7.577 |
| GSM2131588 | prostate cancer | N.A. | N.A. | N.A. | CaP | 7.974 |
| GSM2131589 | prostate cancer | N.A. | N.A. | N.A. | CaP | 6.563 |
| GSM2131590 | prostate cancer | N.A. | N.A. | N.A. | CaP | 7.610 |
| GSM2131591 | prostate cancer | N.A. | N.A. | N.A. | CaP | 7.234 |
| GSM2131592 | prostate cancer | N.A. | N.A. | N.A. | CaP | 8.305 |
| GSM2131593 | prostate cancer | N.A. | N.A. | N.A. | CaP | 7.754 |
| GSM2131594 | prostate cancer | N.A. | N.A. | N.A. | advanced CaP | 8.181 |
| GSM2131595 | prostate cancer | N.A. | N.A. | N.A. | CaP | 7.353 |
| GSM2131596 | prostate cancer | N.A. | N.A. | N.A. | CaP | 7.536 |
| GSM2131597 | prostate cancer | N.A. | N.A. | N.A. | advanced CaP | 7.470 |
| GSM2131598 | prostate cancer | N.A. | N.A. | N.A. | CRPC | 7.578 |
| GSM2131599 | prostate cancer | N.A. | N.A. | N.A. | CRPC | 6.935 |
| GSM2131600 | prostate cancer | N.A. | N.A. | N.A. | advanced CaP | 7.590 |
| GSM2131601 | prostate cancer | N.A. | N.A. | N.A. | CRPC | 8.028 |
| GSM2131602 | prostate cancer | N.A. | N.A. | N.A. | advanced CaP | 6.545 |
| GSM2131603 | prostate cancer | N.A. | N.A. | N.A. | advanced CaP | 8.105 |
| GSM2131604 | prostate cancer | N.A. | N.A. | N.A. | advanced CaP | 7.339 |
| GSM2131605 | prostate cancer | N.A. | N.A. | N.A. | advanced CaP | 8.000 |
| GSM2131606 | prostate cancer | N.A. | N.A. | N.A. | CRPC | 7.778 |
| GSM2131607 | prostate cancer | N.A. | N.A. | N.A. | CRPC | 8.785 |
| GSM2131608 | prostate cancer | N.A. | N.A. | N.A. | CRPC | 8.240 |

Supplementary Table VII. Patients glycemia after ADT.


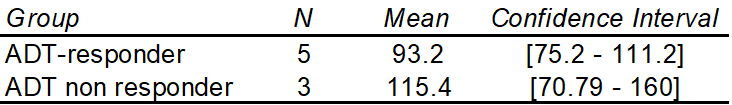

Supplement: Supplementary file 2 — Supplementary material [file 41419_2025_8128_MOESM2_ESM.docx]
