# Supplementary material for: TXNIP upregulation controls metabolism and cell cycle during androgen deprivation therapy in prostate cancer
Source: Cell Death Dis. 2025 Nov 10;16(1):817. doi: 10.1038/s41419-025-08128-4 (PMC12603336; doi:10.1038/s41419-025-08128-4)

Alcon-Rodriguez *et al*, 2025 – Uncropped Western blots

Fig 1J

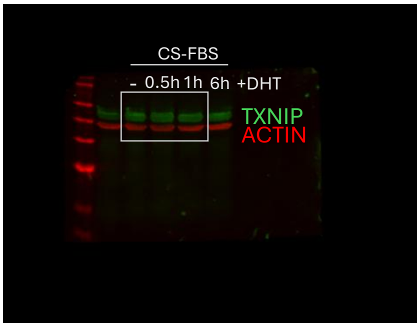

Fig 2A

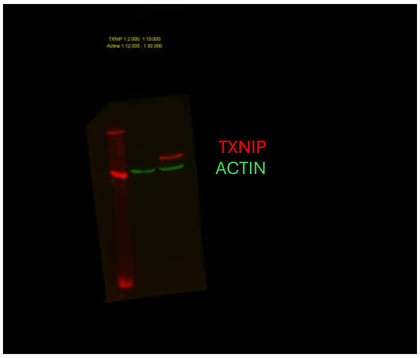

Fig 3K

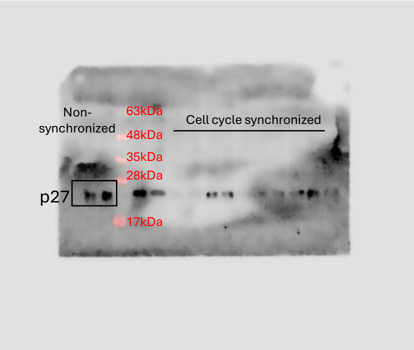

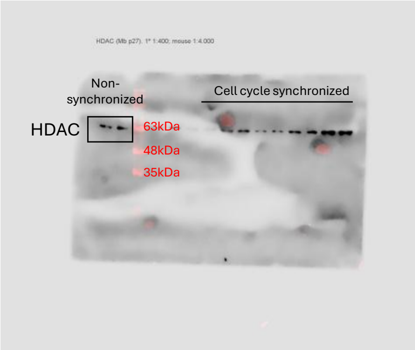

Fig S1A

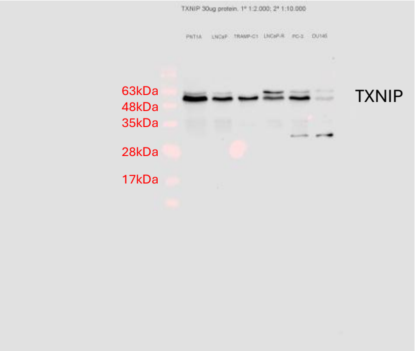

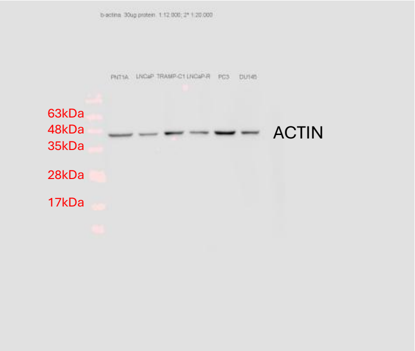

Fig S3D

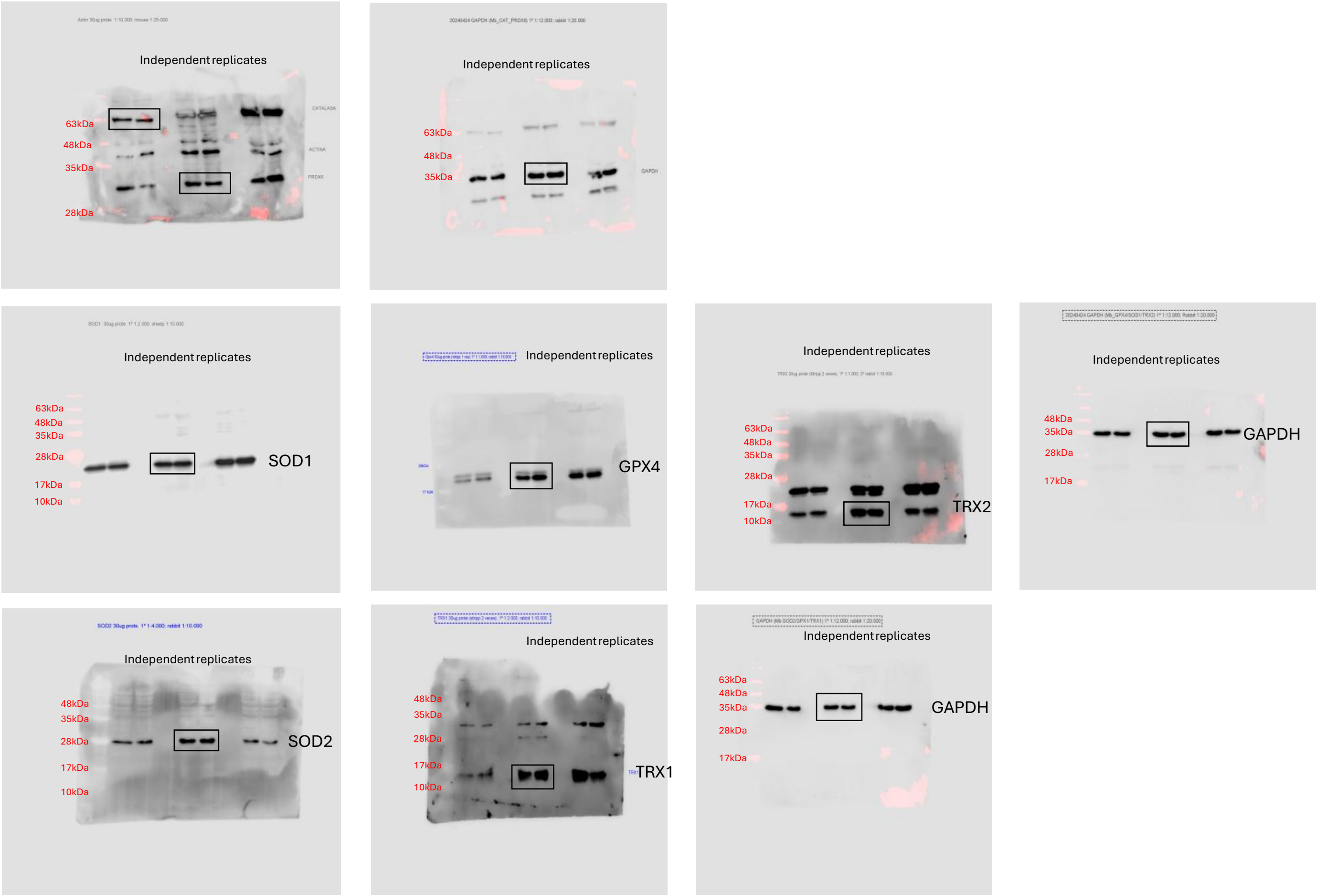

Fig S4A

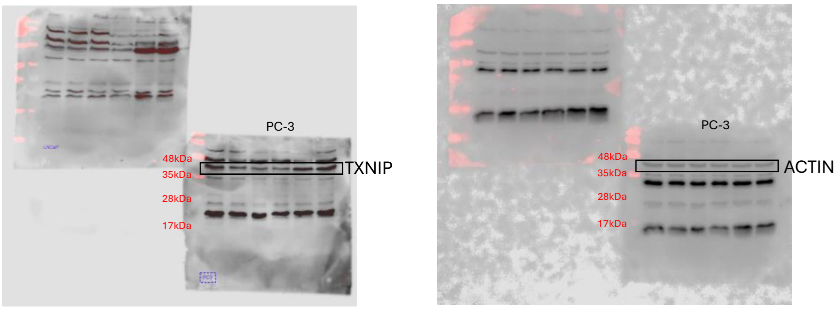

Fig S4J

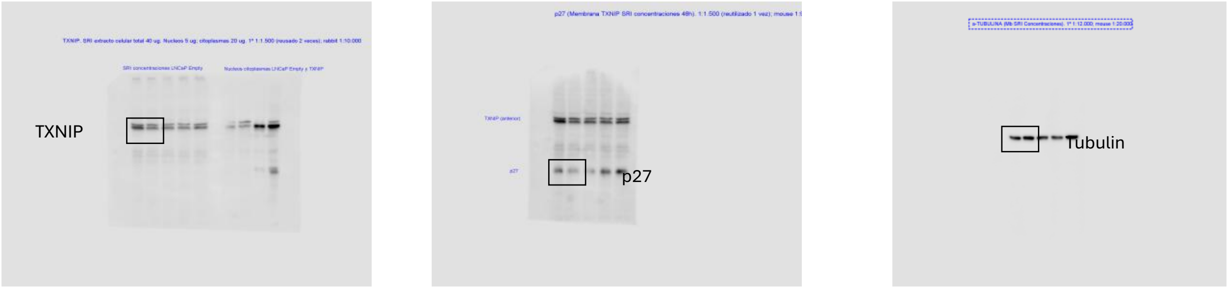

Fig S4L

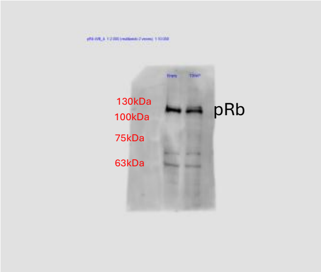

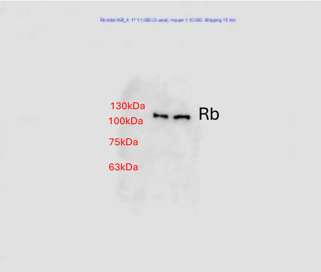

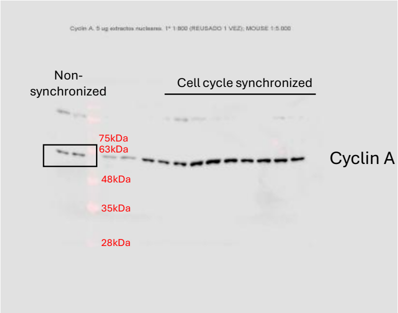

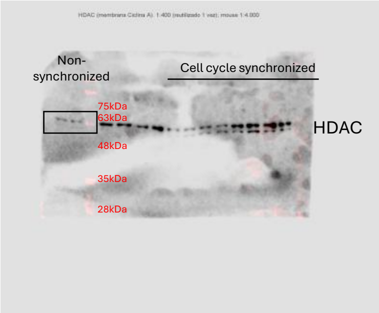

Fig S5A

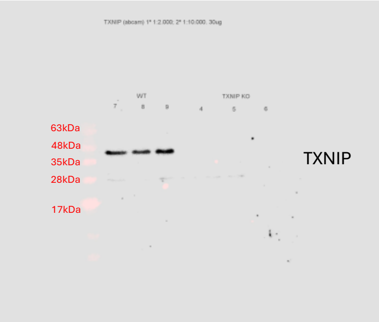

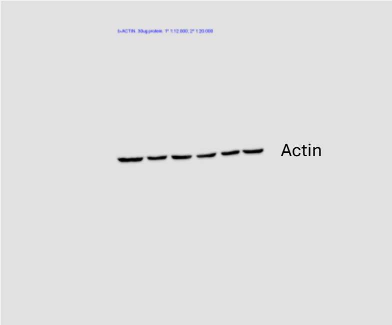

Supplement: Supplementary file 3 — Related Manuscript File [file 41419_2025_8128_MOESM3_ESM.pdf]
